# Cross-validation pitfalls when selecting and assessing regression and classification models

**DOI:** 10.1186/1758-2946-6-10

**Published:** 2014-03-29

**Authors:** Damjan Krstajic, Ljubomir J Buturovic, David E Leahy, Simon Thomas

**Affiliations:** 1 Research Centre for Cheminformatics, Jasenova 7, 11030 Beograd, Serbia; 2 Laboratory for Molecular Biomedicine, Institute of Molecular Genetics and Genetic Engineering, University of Belgrade, Vojvode Stepe 444a, 11010 Beograd, Serbia; 3 Clinical Persona Inc, 932 Mouton Circle, East Palo Alto, CA 94303 USA; 4 Molplex Pharmaceuticals, Alderly Park, Macclesfield SK10 4TF UK; 5 Cyprotex Discovery Ltd, 15 Beech Lane, Macclesfield, SK10 2DR UK

## Abstract

**Background:**

We address the problem of selecting and assessing classification and regression models using cross-validation. Current state-of-the-art methods can yield models with high variance, rendering them unsuitable for a number of practical applications including QSAR. In this paper we describe and evaluate best practices which improve reliability and increase confidence in selected models. A key operational component of the proposed methods is cloud computing which enables routine use of previously infeasible approaches.

**Methods:**

We describe in detail an algorithm for repeated grid-search V-fold cross-validation for parameter tuning in classification and regression, and we define a repeated nested cross-validation algorithm for model assessment. As regards variable selection and parameter tuning we define two algorithms (repeated grid-search cross-validation and double cross-validation), and provide arguments for using the repeated grid-search in the general case.

**Results:**

We show results of our algorithms on seven QSAR datasets. The variation of the prediction performance, which is the result of choosing different splits of the dataset in V-fold cross-validation, needs to be taken into account when selecting and assessing classification and regression models.

**Conclusions:**

We demonstrate the importance of repeating cross-validation when selecting an optimal model, as well as the importance of repeating nested cross-validation when assessing a prediction error.

**Electronic supplementary material:**

The online version of this article (doi:10.1186/1758-2946-6-10) contains supplementary material, which is available to authorized users.

## Background

Allen [1], Stone [2] and Geisser [3], independently introduced cross-validation as a way of estimating parameters for predictive models in order to improve predictions. Allen [1] proposed the PRESS (Prediction Sum of Squares) criteria, equivalent to leave-one-out cross-validation, for problems with selection of predictors and suggested it for general use. Stone [2] suggested the use of leave-one-out cross-validation for estimating model parameters and for assessing their predictive error. It is important to note that Stone [2] was the first to clearly differentiate between the use of cross-validation to select the model (“cross-validatory choice”) and to assess the model (“cross-validatory assessment”). Geisser [3] introduced the Predictive Sample Reuse Method, a method equivalent to V-fold cross-validation, arguing that it improves predictive performance of the cross-validatory choice, at a cost of introducing pseudo-randomness in the process. Since then, cross-validation, with its different varieties, has been investigated extensively and, due to its universality, gained popularity in statistical modelling.

In an ideal situation we would have enough data to train and validate our models (training samples) and have separate data for assessing the quality of our model (test samples). Both training and test samples would need to be sufficiently large and diverse in order to be represenatitive. However such data rich situations are rare in life sciences, including QSAR. A major problem with selection and assessment of models is that we usually only have information from the training samples, and it is therefore not feasible to calculate a test error. However, even though we cannot calculate the test error, it is possible to estimate the expected test error using training samples. It can be shown that the expected test error is the sum of irreducible error, squared bias and variance (Hastie *et al.*[4] Eq 7.9). Furthermore, Hastie *et al.*[4] show the interplay between bias, variance and model complexity in detail. Usually, complex models have small bias and large variance, while simple models have large bias and small variance. We are looking for practically useful trade-offs between bias and variance, for example by minimizing the sum of squared bias and variance.

The model selection process does not require exact computation of various models’ complexity, which is often impossible, but only their relative ranking, which is usually feasible. Hastie *et al.*[4] define effective degrees of freedom and use it as the measure of model complexity. For example, when selecting a model with the k-nearest neighbourhood method, we don’t need to know that the effective degrees of freedom is *N/k*, where *N* is the number of samples. However, for the ranking of models, it is required to understand that the number of neighbours *k* is inversely related to the model complexity.

Hastie *et al.*[4] devote a whole chapter in their book to various methods of selecting and assessing statistical models. In this paper we are particularly interested in examining the use of cross-validation to select and assess classification and regression models. Our aim is to extend their findings and explain them in more detail.

Methodological advances in the last decade or so have shown that certain common methods of selecting and assessing classification and regression models are flawed. We are aware of the following cross-validation pitfalls when selecting and assessing classification and regression models:

- Selection of variables prior to, and not within, cross-validation.
- Selection of model based on performance of a single cross-validation.
- Reporting a cross-validation error as an estimate of error.
- Reporting a single nested cross-validation error as an estimate of error.

We demonstrate the effects of the above pitfalls either by providing references or our own results. We then formulate cross-validation algorithms for model selection and model assessment in classification and regression settings which avoid the pitfalls, and then show results of applying these methods on QSAR datasets.

The contributions of this paper are as follows. First, we demonstrate the variability of cross-validation results and point out the need for repeated cross-validation. Second, we define repeated cross-validation algorithms for selecting and assessing classification and regression models which deliver robust models and report the associated performance assessments. Finally, we propose that advances in cloud computing enable the routine use of these methods in statistical learning.

## Methods

### Repeated cross-validation

In V-fold cross-validation we divide the dataset pseudo randomly into V folds, and a statistical model is refit V times with the cases of each fold withheld in turn from the training set. We analysed the variation in the prediction performance that results from choosing a different split of the data. As far as we are aware, the value and importance of repeated cross-validation has not been extensively explored and discussed in the literature partially, we believe, due to the associated computational costs. To quantify the variation, we repeated cross-validation 50 times and estimated the resulting distribution of the performance statistics.

### Stratified cross-validation

In stratified V-fold cross-validation the output variable is first stratified and the dataset is pseudo randomly split into V folds making sure that each fold contains approximately the same proportion of different strata. Breiman and Spector [5] report no improvement from executing stratified cross-validation in regression settings. Kohavi [6] studied model selection and assessment for classification problems, and he indicates that stratification is generally a good strategy when creating cross-validation folds. Furthermore, we need to be careful here, because stratification *de facto* breaks the cross-validation heuristics.

With a large number of repeated cross-validations our opinion is that the issue of stratification becomes redundant when selecting a model, while for assessing the model it is wise to use stratified cross-validation. We would like to point out that there is no clear consensus regarding the application of stratified cross-validation or any other splitting strategy which takes into account values of the output variable.

Our compromise is not to use stratification for model selection, but to use it for model assessment.

### Parameter tuning with repeated grid-search

We applied cross-validation for parameter tuning in classification and regression problems. How do we choose optimal parameters? In some cases the parameter of interest is a positive integer, such as *k* in k-nearest neighbourhood or the number of components in partial-least squares, and possible solutions are 1,2,3,.. etc. In other cases we need to find a real number within some interval, such as the cost value *C* in linear Support Vector Machine (SVM) or the penalty value *λ* in ridge regression. Chang and Lin [7] suggest choosing an initial set of possible input parameters and performing *grid search cross-validation* to find optimal (with respect to the given grid and the given search criterion) parameters for SVM, whereby cross-validation is used to select optimal tuning parameters from a one-dimensional or multi-dimensional grid. The grid-search cross-validation produces cross-validation estimates of performance statistics (for example, error rate) for each point in the grid. Dudoit and van der Laan [8] give the asymptotic proof of selecting the tuning parameter with minimal cross-validation error in V-fold cross-validation and, therefore, provide a theoretical basis for this approach. However, the reality is that we work in a non-asymptotic environment and, furthermore, different splits of data between the folds may produce different optimal tuning parameters. Consequently, we used *repeated grid-search cross-validation* where we repeated cross-validation *Nexp* times and for each grid point generated *Nexp* cross-validation errors. The tuning parameter with minimal *mean* cross-validation error was then chosen, and we refer to it as the *optimal cross-validatory choice* for tuning parameter. Algorithm 1 is the repeated grid-search cross-validation algorithm for parameter tuning in classification and regression used in this paper:

#### Algorithm 1: parameter tuning with repeated grid-search cross-validation

We have a dataset D which consists of N realisations (Y, X_1_, X_2_,…, X_P_) of one output variable Y and variables X_1_, X_2_,…, X_P_. We have at our disposal a regression or classification model building method F with a tuning parameter vector α. We create a grid of K points α_1_, α_2_,…, α_K_ and wish to find the optimal value among them. Model F predicts either categories for classification or numbers for regression. We have a loss function loss() as a measure of goodness of fit.

1. Repeat the following process *Nexp* times.
  - Divide the dataset D pseudo-randomly into V folds
  - For *I* from 1 to V
    i. Define set L as the dataset D without the *I*-th fold
    ii. Define set T as the *I*-th fold of the dataset D
    iii. For *k* from 1 to K
      1. Build a statistical model f^*k*^ = f(L; α^*k*^)
      2. Apply f^*k*^ on T and store the predictions.
  - For each α value calculate the goodness of fit (loss()) for all elements in D.
2. For each α value calculate the mean of the *Nexp* calculations of losses.
3. Let α’ be the α value for which the average loss is minimal. If there are multiple α values for which the average loss is minimal, then α’ is the one with the lowest model complexity.
4. Select α’ as the optimal cross-validatory choice for tuning parameter and select statistical model f’ = f(D; α’) as the optimal cross-validatory chosen model.

### Nested cross-validation for model assessment

We analysed cross-validation methods for *model assessment*. As Stone [2] pointed out, cross-validation can be used for model selection and for model assessment, but the two tasks require different cross-validation approaches. Even though the process of model selection is different from the process of model assessment, there has been a tendency to report the cross-validation error found for the optimal model during the model selection as the assessed model performance. Varma and Simon [9] report a bias in error estimation when using cross-validation for model selection, and they suggest using “nested cross-validation” as an almost unbiased estimate of the true error. Close examination shows that the “nested cross-validation” defined by Varma and Simon [9] is the same as “cross-validatory assessment of the cross-validatory choice” defined by Stone [2]. Nevertheless, the importance of the paper by Varma and Simon [9] is that they show in practice by how much a cross-validation error of a cross-validatory chosen model can be biased, i.e. too optimistic. Therefore, we applied stratified nested cross-validation to reduce bias of the resulting error rate estimate.

We refer to procedure of selecting optimal cross-validatory chosen model with pre-defined grid, number of folds and number of repeats as the *cross-validation protocol*. It is very similar to Stone’s [2]*cross-validatory choice*, but more specific. Using Stone’s [2] terminology we can say that the nested cross-validation is the cross-validation assessment of large-sample performance of a model M chosen by a specific cross-validation protocol P. To emphasize the fact that the nested cross-validation estimate depends on the cross-validation protocol, we refer to it as the *P-estimate* of large-sample performance of model M.

We would like to point out that the “wrapper algorithm” as defined by Varma and Simon [9] is similar to our cross-validation protocol, although our definition is more specific. The “estimation plan” as defined by Dudoit and van der Laan [8] is almost identical to our cross-validation protocol, the only difference being that we specify repetition.

We also demonstrate that single stratified nested cross-validation errors can vary substantially between different partitionings of the training dataset, and therefore used repeated stratified nested cross-validation. Algorithm 2 is the general algorithm for repeated stratified nested cross-validation.

#### Algorithm 2: repeated stratified nested cross-validation

1. Cross-validation protocol *P* is to use *Nexp1* repeated V1-fold cross-validation with a grid of K points α_1_, α_2_,…, α_K._ Designate by M the model chosen by application of the cross-validation protocol *P.*
2. Repeat the following process *Nexp2* times.
  - Stratify the output variable Y.
  - Divide the dataset D pseudo-randomly into V2 folds making sure that each fold contains the same proportion of each of the Y strata.
  - For *I* from 1 to V2
    i. Define set L as the dataset D without the *I*-th fold
    ii. Define set T as the *I*-th fold of the dataset D
    iii. Apply the cross-validation protocol to select model f’, i.e. use *Nexp1* repeated V1-fold cross-validation with a grid of K points α_1_, α_2_,…, α_K_ to find an optimal cross-validatory chosen model f’ on dataset L.
    iv. Apply f’ on T
  - Calculate loss() for all elements of D. We refer to it as the nested cross-validation error.
3. The interval between the minimum and maximum of *Nexp2* nested cross-validation errors is the P-estimated interval of the large-sample error of model M. The mean of *Nexp2* nested cross-validation errors is the P-estimate of the large-sample error of the model M.

We are not aware of any research finding which suggests that number of folds in the outer cross-validation loop (V2) and number of folds in the inner cross-validation loop (V1) need to be the same or different. Similarly, the number of repeats of the nested cross-validation may or may not be equal to the number of repeats of the cross-validation. We used nested cross-validation with V1 = V2 = 10 and Nexp1 = Nexp2 = 50.

In addition to mean nested cross-validation error we reported the minimum and maximum nested cross-validation errors because the variability is such that reporting a single error value may be misleading.

### Variable selection and parameter tuning

The relationship between variable selection and cross-validation was first independently tackled by Allen [1] and Stone [2]. Unfortunately the importance of selecting variables within, and not prior to, cross-validation was widely missed. Ambroise and McLachlan [10] showed how results are biased when selection of variables is done prior to cross-validation. Hastie *et al.*[4] in chapter 7.10.2 of their book defined the correct way to carry out cross-validation as follows:

1. *Divide the samples into K cross-validation folds (groups) at random.*
2. *For each fold k = 1,2,..,K*
  - *Find a subset of “good” predictors that show fairly strong (univariate) correlation with the class labels, using all of the samples except those in fold k.*
  - *Using just this subset of predictors, build a multivariate classifier, using all of the samples except those in fold k.*
  - *Use the classifier to predict the class labels for the sample in fold k.*

*The error estimates from step 2(c) are then accumulated over all K folds, to produce the cross-validation estimate of the prediction error.*

However, Hastie *et al.*[4] did not elaborate any further. As far as we are aware, there are two different ways to implement the above correctly, and we explain each in detail below.

When selecting variables and parameter tuning, our goal is to select the optimal number of variables and the optimal parameter values. Here, again, we can view this as a hyper-parameter optimisation problem and apply grid search. Cross-validation would be used for selecting the number of variables (n) and for tuning parameters (α) from a multi-dimensional grid (n, α), where n ∈ (1, 2,…, P) and α ∈ (α_1_, α_2_,…, α_K_). This requires only one cross-validation loop because it treats each point in the multi-dimensional grid independently. We use the same notation as before with an additional variable selection method S, which for the sake of simplicity only takes two input parameters (number of variables to select and the dataset) and returns a new dataset with only the selected variables.

#### Algorithm 3: repeated grid-search cross-validation for variable selection and parameter tuning

- Repeat the following process *Nexp* times.
  - Divide the dataset D pseudo-randomly into V folds
  - For *I* from 1 to V
  - Define set L as the dataset D without the *I*-th fold
  - Define set T as the *I*-th fold of the dataset D
  - For *p* from 1 to P
  - For each point in the grid (n, α) calculate loss() for all elements of D.
- L’ = S(L; *p*); Define set L’ as set L with only *p* selected variables.
- Define T’ as set T with only *p* selected variables as in L’.
- For *k* from 1 to K
- Build a statistical model f’ = f(L’; α^*k*^)
- Apply f’ on T’ and store predictions.
- For each point in the grid (n, α) calculate average loss.
- Define the pair (p’, α’) with minimal average loss as the optimal pair of number of selected variables and parameter values.
- D’ = S(D; p’); define set D’ as D with only p’ selected predictor variables.
- Select statistical model f’ = f(D’; α’) as the optimal model.

### Double cross-validation

Stone [2] suggested an algorithm under the name “double cross-validation” which involves an additional (internal) cross-validation for parameter tuning for each set of selected variables. As it contains an external and internal cross-validation similar to nested cross-validation, we have found that terms “double cross-validation” and “nested cross-validation” have been used in the literature with different meanings. We use the term “nested cross-validation” as did Varma and Simon [9], meaning the model assessment procedure, and “double cross-validation”, as did Stone [2], meaning the model selection procedure where variables are selected in addition to parameter tuning. Even though we are not using double cross-validation, we consider it to be important to describe it in our context.

#### Algorithm 4: double cross-validation

The double cross-validatio algorithm consists of two steps.

Step 1. Select number of variables

1. Divide the dataset D pseudo-randomly into V1 folds
2. For *I* from 1 to V1
  - Define set L as the dataset D without the *I*-th fold
  - Define set T as the *I*-th fold of the dataset D
  - For *p* from 1 to P
    i. L’ = S(L; *p*); Define set L’ as set L with only *p* selected predictor variables.
    ii. Define T’ as set T with only *p* selected predictors as in L’.
    iii. Divide the dataset L’ pseudo-randomly into V2 folds
    iv. For *J* from 1 to V2
      1. Define set LL’ as the dataset L’ without *J*-th fold
      2. Define set TL’ as the *J*-th fold of the dataset L’
      3. For *k* from 1 to K
        - Build a statistical model f’ = f(LL’; α^*k*^)
        - Apply f’ on TL’ and store predictions.
    v. For each α value calculate the loss() for all elements in L’.
    vi. Define α’ as α value for which the loss function is minimal.
    vii. Build a statistical model f’ = f(L’; α’)
    viii. Apply f’ on T and store predictions
3. For each number of selected variables calculate loss() for all elements of D.
4. Define p’ as the number of selected variables for which the loss() is minimal.
5. Select p’ as the optimal cross-validatory choice of number of selected variables.

Step 2. Select tuning parameter

1. D’ = S(D; p’); define set D’ as set D with only p’ selected predictor variables.
2. Divide the dataset D’ pseudo-randomly into V folds
3. For *I* from 1 to V
  - Define set L’ as the dataset D’ without *I*-th fold
  - Define set T’ as the *I*-th fold of the dataset D’
  - For *k* from 1 to K
    i. Build a statistical model f’ = f(L’; α^*k*^)
    ii. Apply f’ on T’ and store predictions.
4. For each α value calculate the loss() for all elements in D’
5. Let α’ be α value for which the loss is minimal.
6. Select α’ as the optimal cross-validatory choice of tuning parameter and select statistical model f’ = f(D’; α’) as the optimal cross-validatory chosen model.

We are not aware of any research that suggests using grid-search in favour of double cross-validation or vice versa. However, in our opinion, double cross-validation as defined above should not be used when parameters used for tuning affect model complexity. For example, if we use a variable selection method and k-nearest neighbourhood, then both the number of selected variables and number of neighbours, k, directly affect model complexity. Therefore, in step 1 in the external loop we might choose different k for different L’ and for a fixed number of variables end up averaging over models with different model complexities. This cannot happen with grid-search cross-validation, because each point in the grid has a fixed number of selected variables and a fixed number of neighbours, k. Furthermore, each point in the grid is treated independently of all others. We used grid-search cross-validation in all experiments.

### Pre-processing

As we mentioned earlier, it is a mistake to select variables prior to cross-validation. However, it is worth noting that unsupervised screening procedures, like removing variables with near zero variance, in our opinion may be executed prior to the cross-validation loop. In our examples we removed variables if the ratio of the most common value to the second most common value is higher than 95/5 = 19 or if the percentage of distinct values out of the number of total samples is less than 10. Furthermore, we removed variables that are linear combination of other variables. In a ‘complete’ dataset with all possible entries the removed variables may well have more variability or may not be linear combinations of other variables, but in our limited samples they either don’t have additional information (for linear combinations) or cannot be used in cross-validation (variables with near zero variation).

The issue of removing variables prior to model building is, however, not without contention. Zhu *et al.*[11] focus on the bias that arises when a full data set is not available compared to the prediction rule that is formed by working with top-ranked variables from the full set.

### Data sets

In this section, we report results of applying Algorithms 1–3 on seven QSAR datasets. Table 1 shows the summary of the datasets. Note that in this Section we sometimes use the term “descriptor” instead of “input variable” as is common in QSAR. We have used the following publicly available datasets from the QSARdata R package [12]:

- *AquaticTox* contains negative log of toxic activity for 322 compounds. It was described and compiled by He and Jurs [13]. The package contains several sets of descriptors for this problem. We chose to use two dimensional MOE descriptors as an example, because when compared to other descriptor sets it generated better models (results not shown). There are 220 MOE 2D descriptors for each compound. However, during pre-processing we removed 30 descriptors with near zero variation and 6 descriptors that were linear combinations of others, leaving 184 descriptors for model building.
- *bbb2* contains blood–brain barrier categories (“crossing” or “not crossing”) for 80 compounds from Burns andWeaver [14]. There are 45 compounds categorised as “crossing” and 35 compounds as “not crossing”. The package contains several sets of descriptors for this problem. We chose to use LCALC descriptors as an example, because when compared to other descriptor sets it generated better models (results not shown). We had to remove chloramphenicol from the dataset because LCALC descriptors were not provided for it. There are 23 LCALC descriptors for each compound. During pre-processing we removed descriptor LCALC_NDA as it was a linear combinations of others, leaving 22 descriptors for model building.
- *caco* contains permeability categories (“low”,“medium”,“high”) for 3796 compounds from Pham-The *et al.*[15]. There are 377 compounds categorised as “low”, 2029 compounds as “medium” and 1390 compounds as “high”. The package contains several sets of descriptors for this problem. As this is the only multi category dataset, we chose to use two sets of descriptors (PipelinePilotFP and QuickProp), because when compared to other descriptor sets they generated better models (results not shown). There are 5401 PipelinePilotFP descriptors for each compound. During pre-processing we removed 4503 descriptors with near zero variation and 519 descriptors that were linear combinations of others, leaving 379 PipelinePilotFP descriptors for model building. There are 51 QuickProp descriptors for each compound. During pre-processing we removed 4 descriptors with near zero variation, leaving 47 QuickProp descriptors for model building.
- *MeltingPoint* containts melting points for 4126 compounds used for model building in Karthikeyan *et al.*[16]. In the QSARdata package there is one set of 202 descriptors. During pre-processing we removed 11 descriptors with near zero variation and 22 descriptors that were linear combinations of others, leaving 169 descriptors for model building.
- *Mutagen* contains mutagenicity categories (“mutagen” or “nonmutagen”) for 4335 compounds from Kazius *et al.*[17]. There are 2400 compounds categorised as “mutagen” and 1935 compounds as “nonmutagen”. In the package there is one set of 1579 descriptors. During pre-processing we removed 281 descriptors with near zero variation and 15 descriptors that were linear combinations of others, leaving 1283 descriptors for model building.
- *PLD* contains phospholipidosis categories (“inducer” or “noninducer”) for 324 compounds from Goracci *et al.*[18]. There are 124 compounds categorised as “inducer” and 200 compounds as “noninducer”. The package comes with several sets of descriptors for this problem. We chose to use PipelinePilotFP, because when compared to other descriptor sets it generated better models (results not shown). There are 2862 PipelinePilotFP descriptors for each compound. During pre-processing we removed 2183 descriptors with near zero variation and 371 descriptors that were linear combinations of others, leaving 308 descriptors for model building.

**Table 1:** **Seven QSAR datasets**

Dataset	Output	Number of compounds	Number of descriptors after preprocessing
AquaticTox	Numeric	322	184
bbb2	2 Categories	79	22
Caco-PipelinePilotFP	3 Categories	3796	379
Caco-QuickProp	3 Categories	3796	47
MeltingPoint	Numeric	4126	169
Mutagen	2 Categories	4335	1283
PLD	2 Categories	324	308

Summary of 7 QSAR datasets.

### Methods for prediction

In our examples we apply ridge regression and partial-least squares (PLS) on regression problems, while for classification problems we use ridge logistic regression and linear SVM coupled with Pearson’s rank based variable selection. We use sum of squared residuals and proportion misclassified as the loss functions for regression and classification, respectively.

The process of ranking and selecting *P* variables using Pearson’s correlation is as follows. The Pearson’s correlation coefficient is calculated between each input variable Xi and the output variable Y. The absolute values of the coefficients are sorted in descending order and the first *P* variables are selected. The method is quick and in our experience works well with the SVM classification method.

SVM is a widely used technique in solving classification problems. SVM performs classification by constructing an N-dimensional hyper plane that optimally separates the data into two categories. SVM is usually applied in conjunction with a kernel function, which is used to transform input data into a higher-dimensional space where the construction of the hyperplane is easier. There are four basic SVM kernels: linear, polynomial, Radial Basis Function (RBF), and sigmoid. For the sake of simplicity we use linear SVM, which requires a parameter C (cost) to be supplied. We searched for the optimal model with values for C of 0.5, 1, 2, 4, 8, and 16. We used the R package e1071 [19] for building SVM models.

Hoerl and Kennard [20] proposed ridge regression, a penalized least squares regression, to achieve better predictions in the presence of multicolinearity of predictors. In ridge regression the extent of coefficient shrinkage is determined by one parameter, usually referred to as lambda (λ), and it is inversely related to the model complexity. Applying ridge regression tends to improve prediction performance but it results in all small, but non-zero, regression coefficients. Friedman *et al.*[21] developed a fast algorithm for fitting generalised linear models with various penalties, and we used their glmnet R package [22] to apply ridge regression and ridge logistic regression for classification purposes. Typical usage is to let the glmnet function compute its own array of lambda values based on nlambda (number of lambda values – default is 100) and lambda.min.ratio (ratio between the maximum and minimum lambda value). We searched for the optimal model with nlambda = 100 and lambda.min.ratio = 10^−6^.

PLS was introduced by Wold [23]. The method iteratively creates components, which are linear combination of input variables, with a goal of maximising variance and correlation with the output variable. The idea is to transform the input space of X_1_, X_2_,…, X_P_ variables into a new hyper plane, with low dimensions, such that coordinates of the projections onto this hyper plane are good predictors of the output variable Y. As it is an iterative process, with each newly added component we increase complexity of the model. The method is very popular amongst QSAR modellers due to its simplicity and good results in high-dimensional settings. We searched for the optimal model with a grid of number of components from 1 to 60. We used the R package pls [24] for building PLS models.

## Results and experimental

### Repeated cross-validation

We applied Algorithm1 with Nexp = 50 and V = 10 to the following nine combinations of modelling method and dataset:

1. PLS on AquaticTox
2. Ridge regression on AquaticTox
3. Ridge logistic regression on bbb2
4. Ridge logistic regression on caco-PipelinePilotFP
5. Ridge logistic regression on caco-QuickProp
6. PLS on MeltingPoint
7. Ridge regression on MeltingPoint
8. Ridge logistic regression on Mutagen
9. Ridge logistic regression on PLD

In order to show how the cross-validatory choice of parameter may vary if based on single cross-validation, for all nine cases we found 50 cross-validatory chosen parameters corresponding to 50 single cross-validations. Table 2 shows distributions of optimal cross-validatory chosen parameters for each dataset. It is obvious that the model selected by single cross-validation may have high variance.

**Table 2:** **Distribution of optimal parameters**

PLS on aquaticTox	Number of components	10	11	12	13	14	15			
	Frequency	1	9	9	23	6	2			
Ridge regression on AquaticTox	Lambda	≤0.027	0.035	0.040	0.046	0.053	0.061	0.070	0.081	≥0.093
	Frequency	6	5	7	8	4	6	10	6	2
Ridge logistic regression on bbb2	Lambda	≤0.09	0.10	0.12	0.14	0.16	0.18	0.21	0.24	≥0.28
	Frequency	7	3	4	5	10	6	5	2	8
Ridge logistic regression on caco-PipelinePilotFP	Lambda	<0.0046	0.0046	0.0053	0.0061	0.0070	0.0081	0.0093	0.0107	>0.0107
	Frequency	6	2	2	4	7	12	6	6	5
Ridge logistic regression on caco-QuickProp	Lambda	≤0.018	0.021	0.024	0.028	0.032	0.037	0.042	0.049	≥0.056
	Frequency	7	2	8	7	7	7	4	4	4
PLS on MeltingPoint	Number of components	34-35	36	37-40	41	42-46	47	48-51	57	60
	Frequency	7	7	6	8	7	8	5	1	1
Ridge regression on MeltingPoint	Lambda	≤0.031	0.036	0.042	0.048	0.055	0.063	0.073	0.084	≥0.096
	Frequency	5	1	4	6	5	5	7	10	5
Ridge logistic regression on Mutagen	Lambda	<0.0016	0.0016	0.0018	0.0021	0.0024	0.0031	0.0036	0.0042	>0.0042
	Frequency	7	2	1	6	5	8	4	6	7
Ridge logistic regression on PLD	Lambda	≤0.34	0.34	0.39	0.44	0.67	0.77	0.89	1.02	≥1.17
	Frequency	10	2	3	2	1	5	5	5	19

Distribution of optimal parameters (number of components or lambda values) based on 50 single cross-validations for each pair of method/dataset.

Figures 1, 2, 3, 4, 5, 6, 7, 8 and 9 show for each dataset/method combination the minimum, first quartile, median, third quartile and maximum cross-validated loss () from 50 repeats as a function of the single hyperparameter.

**Figure 1:**
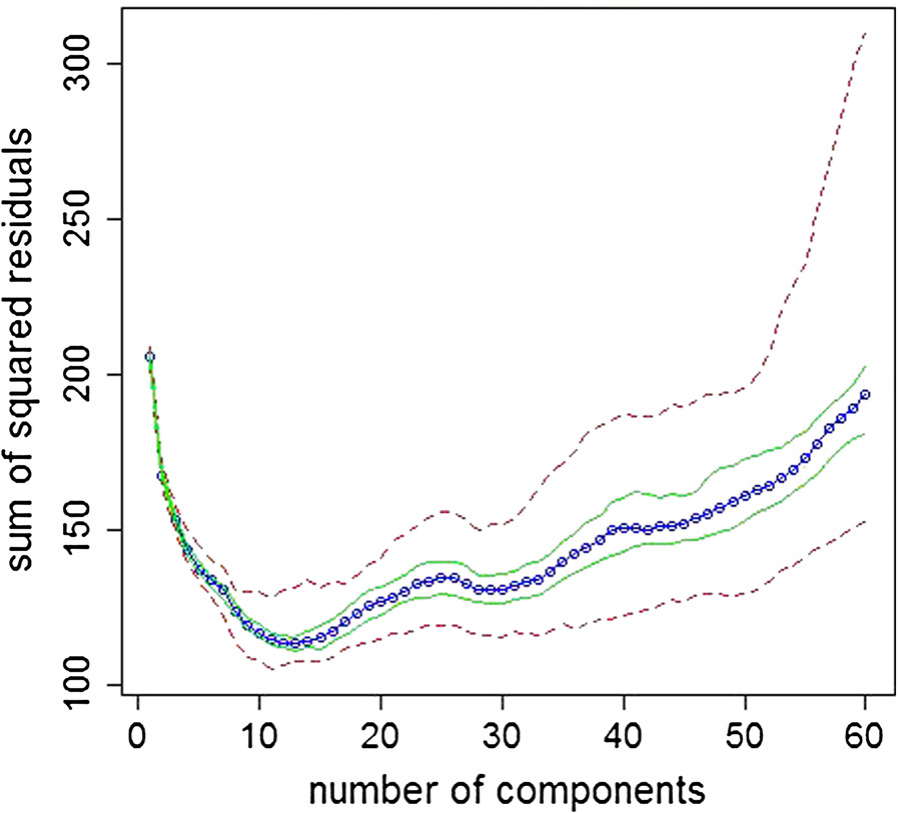
**PLS on AquaticTox (50 repeats 10 fold CV).** Minimum, first quartile, median, third quartile and maximum cross-validated sum of squared residuals from 50 repeats of 10-fold cross-validation of PLS on AquaticTox for number of components from 1 to 60.

**Figure 2:**
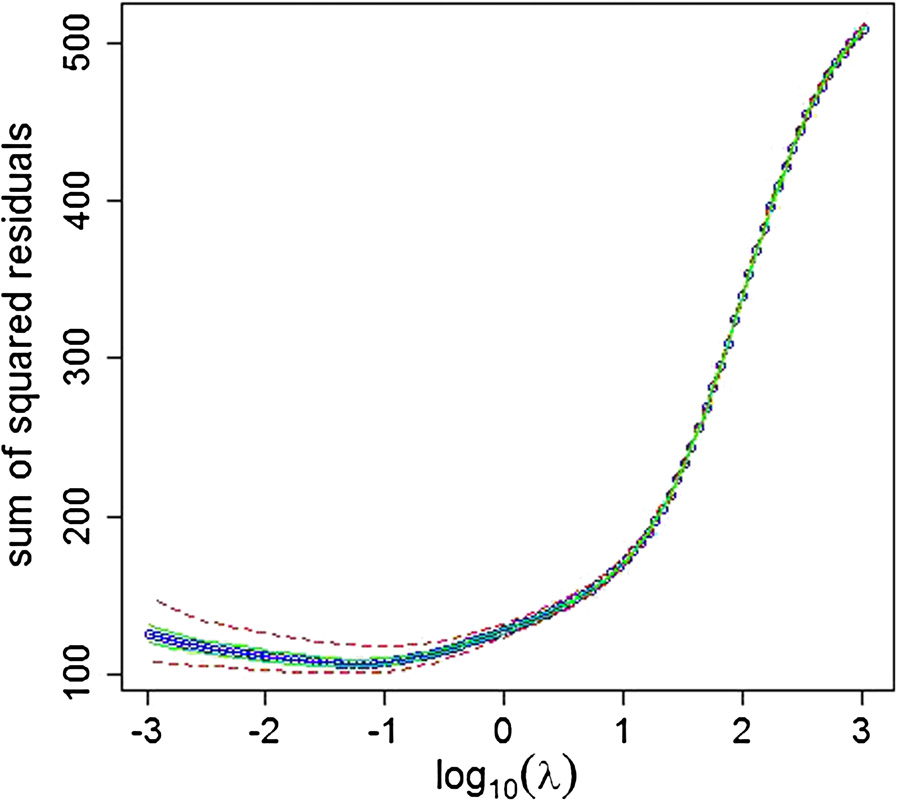
**Ridge regression on AquaticTox (50 repeats 10 fold CV).** Minimum, first quartile, median, third quartile and maximum cross-validated sum of squared residuals from 50 repeats of 10-fold cross-validation of ridge regression on AquaticTox for 100 λ values.

**Figure 3:**
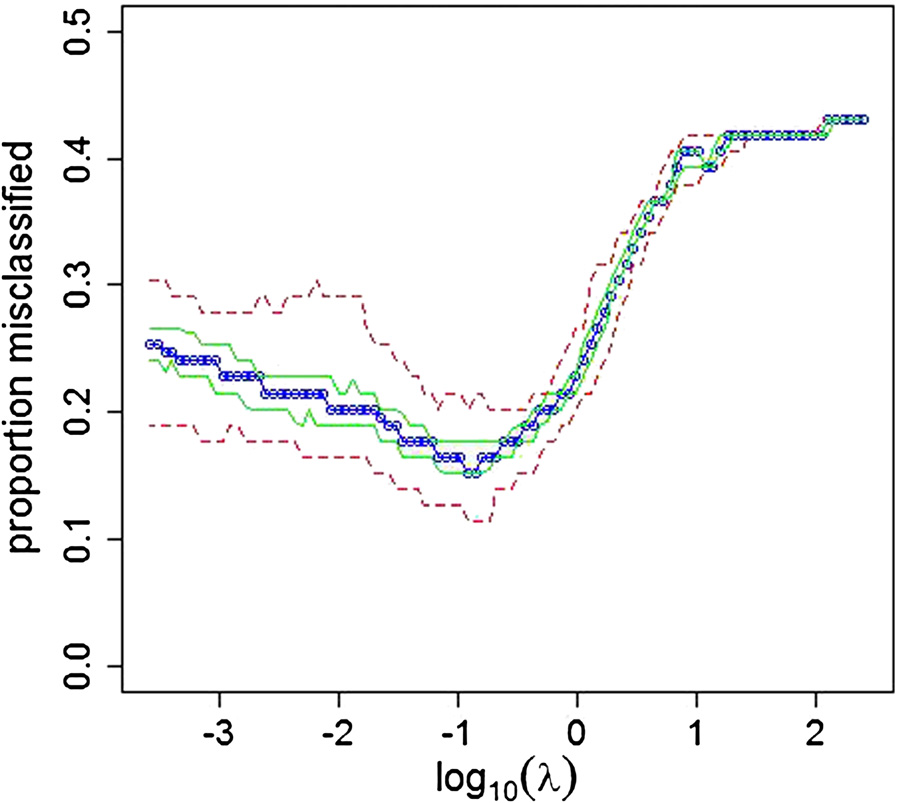
**Ridge logistic regression on bbb2 (50 repeats 10 fold CV).** Minimum, first quartile, median, third quartile and maximum cross-validated proportion misclassified from 50 repeats of 10-fold cross-validation of ridge logistic regression on bbb2 for 100 λ values.

**Figure 4:**
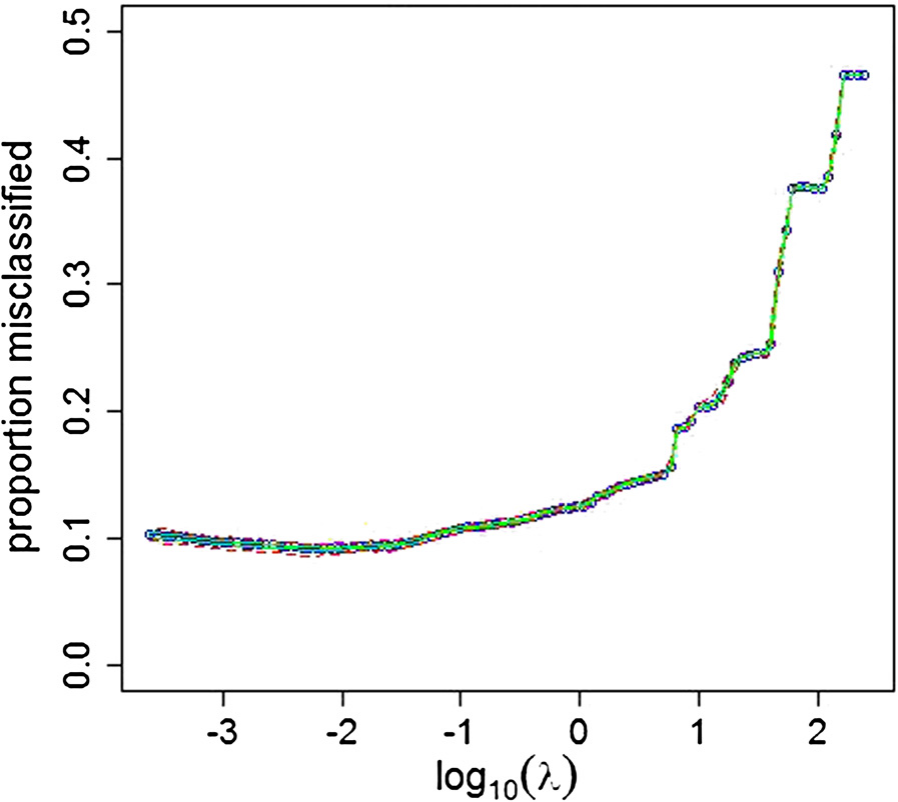
**Ridge logistic regression on caco-PipelinePilotFP (50 repeats 10 fold CV).** Minimum, first quartile, median, third quartile and maximum cross-validated proportion misclassified from 50 repeats of 10-fold cross-validation of ridge logistic regression on caco-PipelinePilotFP for 100 λ values.

**Figure 5:**
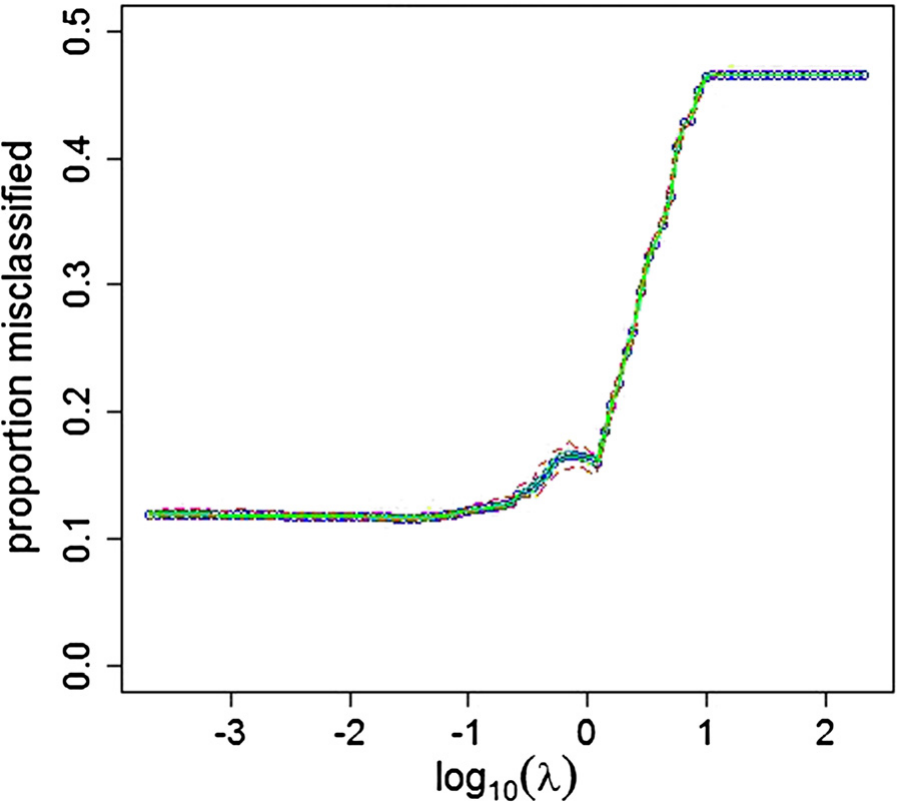
**Ridge logistic regression on caco-QuickProp (50 repeats 10 fold CV).** Minimum, first quartile, median, third quartile and maximum cross-validated proportion misclassified from 50 repeats of 10-fold cross-validation of ridge logistic regression on caco-QuickProp for 100 λ values.

**Figure 6:**
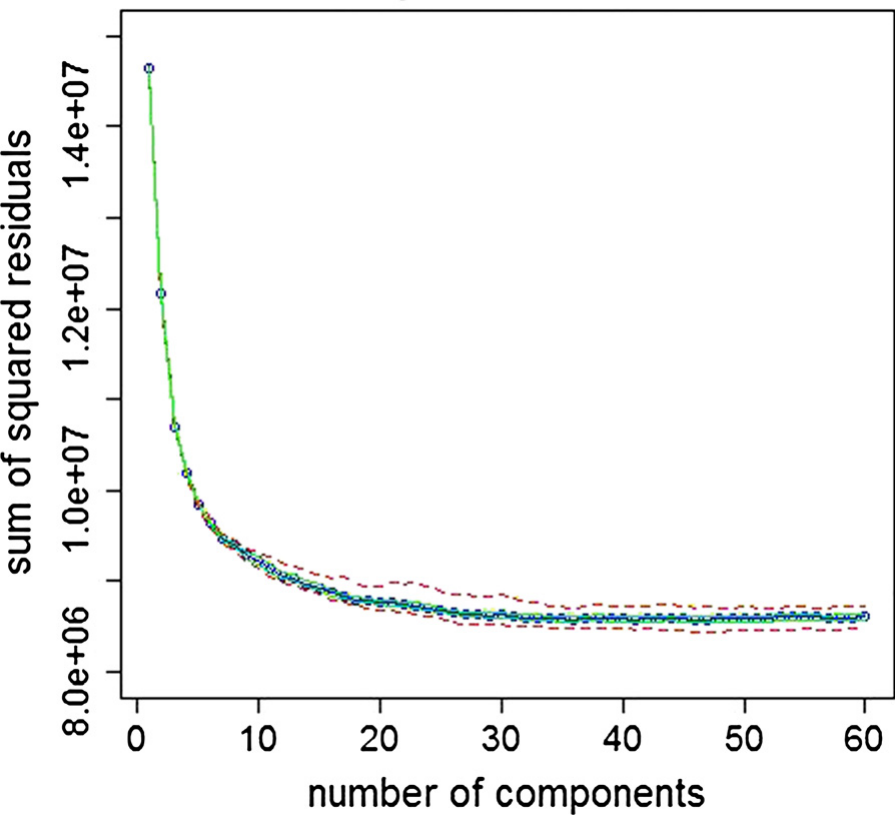
**PLS on MeltingPoint (50 repeats 10 fold CV).** Minimum, first quartile, median, third quartile and maximum cross-validated sum of squared residuals from 50 repeats of 10-fold cross-validation of PLS on MeltingPoint for number of components from 1 till 60.

**Figure 7:**
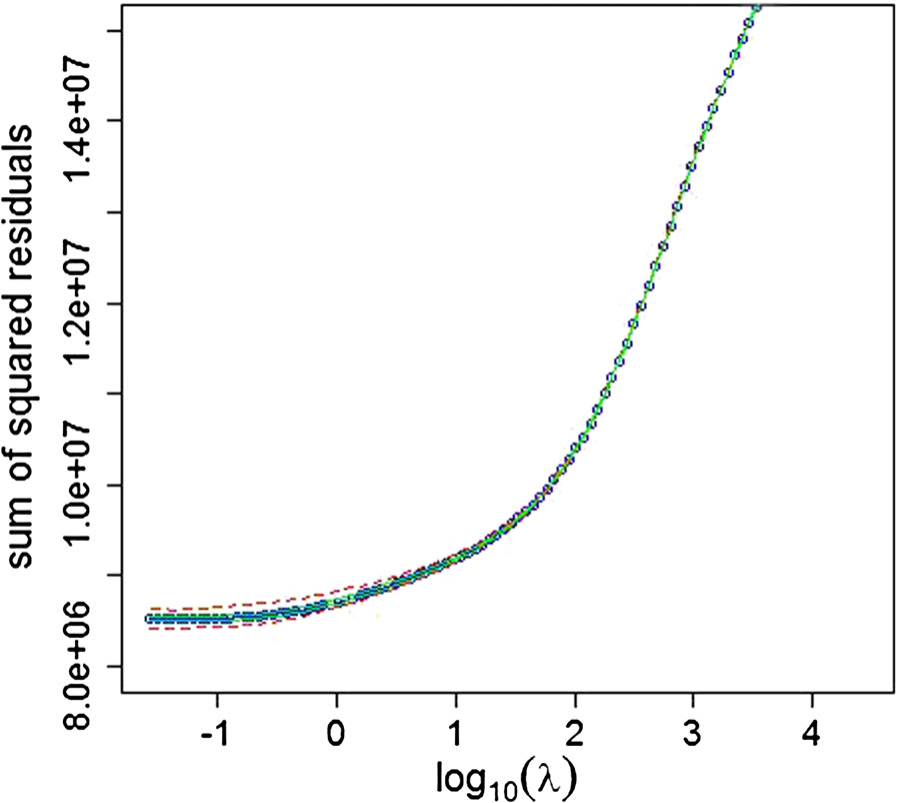
**Ridge regression on MeltingPoint (50 repeats 10 fold CV).** Minimum, first quartile, median, third quartile and maximum cross-validated sum of squared residuals from 50 repeats of 10-fold cross-validation of ridge regression on MeltingPoint for 100 λ values.

**Figure 8:**
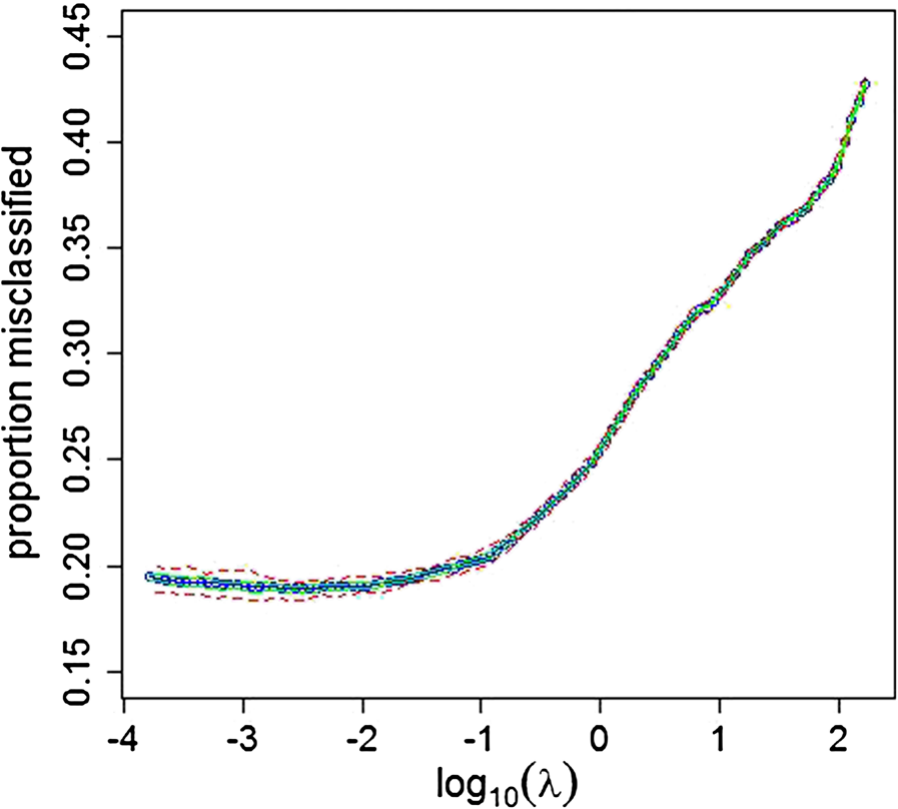
**Ridge logistic regression on Mutagen (50 repeats 10 fold CV).** Minimum, first quartile, median, third quartile and maximum cross-validated proportion misclassified from 50 repeats of 10-fold cross-validation of ridge logistic regression on Mutagen for 100 λ values.

**Figure 9:**
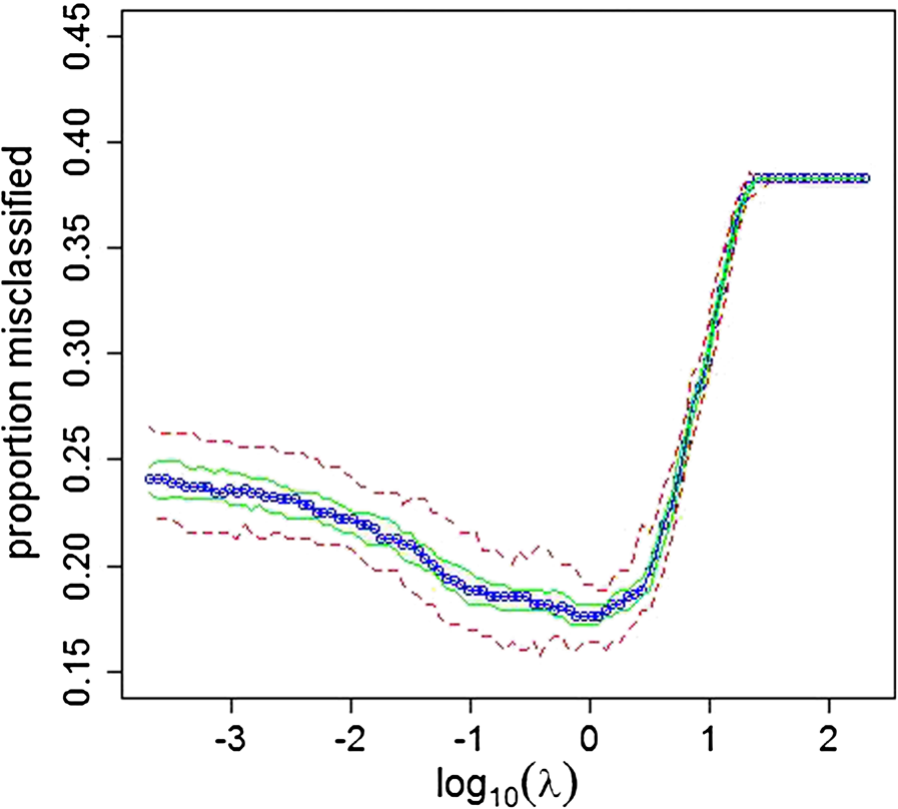
**Ridge logistic regression on PLD (50 repeats 10 fold CV).** Minimum, first quartile, median, third quartile and maximum cross-validated proportion misclassified from 50 repeats of 10-fold cross-validation of ridge logistic regression on PLD for 100 λ values.

### Nested cross-validation

In order to assess the quality of our protocols, which generated the cross-validatory chosen models reported in Table 3, we applied repeated stratified nested cross-validation (Algorithm 2) with Nexp1 = Nexp2 = 50 and V1 = V2 = 10 on the nine dataset/method combinations.

**Table 3:** **Selected optimal cross-validatory chosen models**

Dataset	Model	Lowest average cross_validation loss	Optimal parameter	Min grid value	Max grid value	Grid size
AquaticTox	PLS	0.5948	13	1	60	60
AquaticTox	Ridge	0.5767	0.05325	0.00107	1069.93	100
bbb2	Ridge	0.1689	0.10494	0.00026	260	100
Caco-PipelinePilotFP	Ridge	0.0916	0.008058	0.00025	246	100
Caco-QuickProp	Ridge	0.1162	0.0279	0.00021	211	100
MeltingPoint	PLS	45.5848	47	1	60	60
MeltingPoint	Ridge	45.4370	0.0549	0.02734	27346.2	100
Mutagen	Ridge	0.1889	0.003142	0.00017	168	100
PLD	Ridge	0.1768	1.02431	0.00021	205.81	100

Summary of selected optimal cross-validatory chosen models from nine examples.

Our goal is to show examples of nested cross-validation results and its benefits, and not to analyse why one method or set of descriptors performed better than the other.

**Figure 10:**
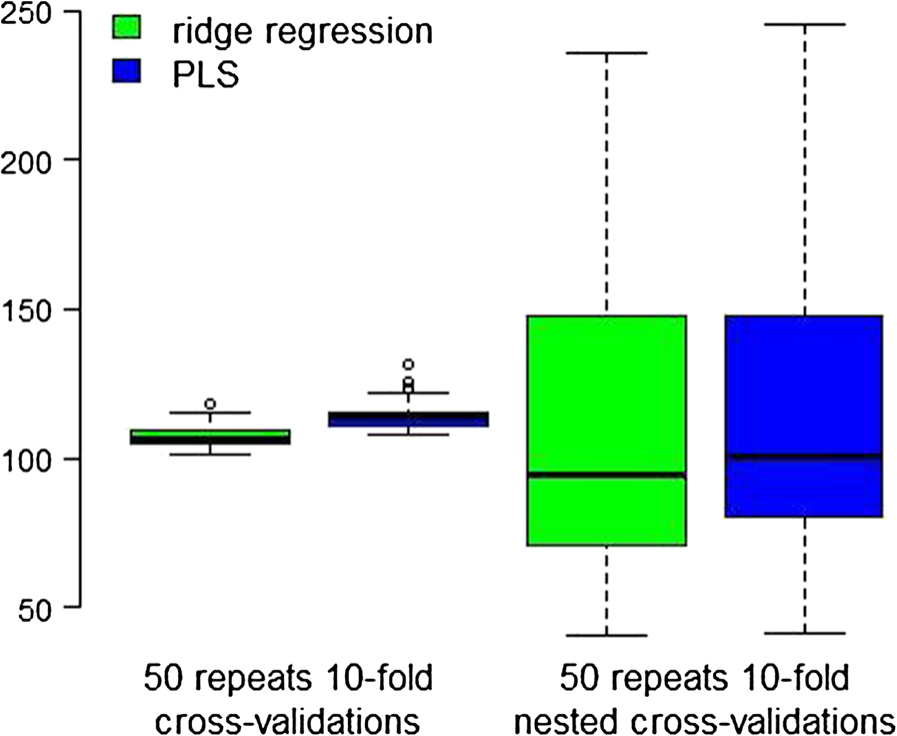
**Cross-validation and nested cross-validation sum of squared residuals for ridge regression and PLS on AquaticTox.** Boxplots of 50 cross-validation sum of squared residuals for ridge regressiona and PLS on AquaticTox and 50 nested cross-validation sum of squared residuals for ridge regressiona and PLS on AquaticTox.

**Figure 11:**
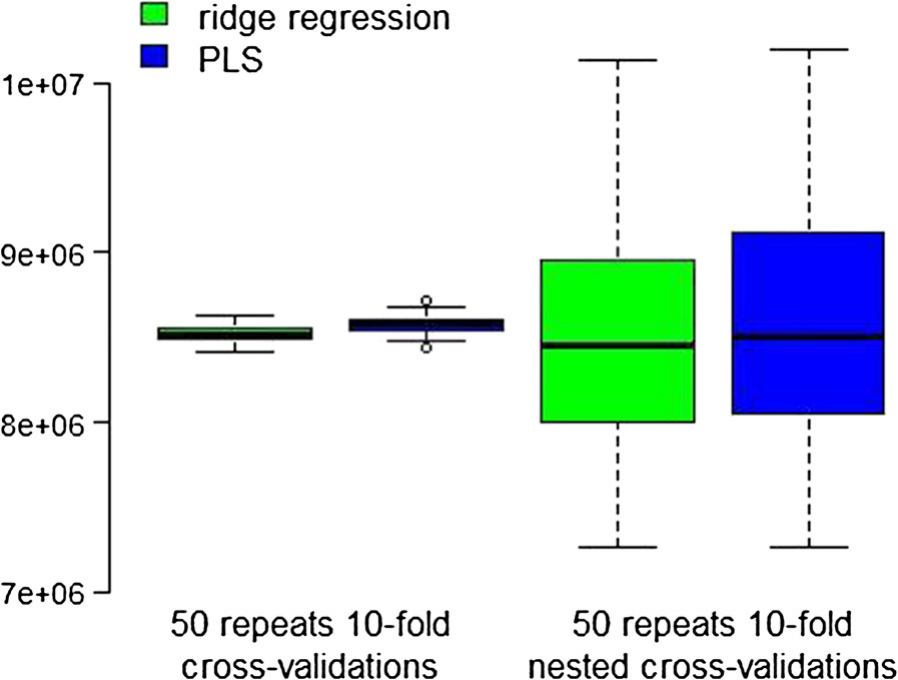
**Cross-validation and nested cross-validation sum of squared residuals for ridge regression and PLS on MeltingPoint.** Boxplots of 50 cross-validation sum of squared residuals for ridge regressiona and PLS on MeltingPoint and 50 nested cross-validation sum of squared residuals for ridge regressiona and PLS on MeltingPoint.

We applied two linear regressions (PLS and ridge) on AquaticTox (Figure 10) and MeltingPoint (Figure 11). Ridge models on average give slightly better error estimates than PLS models. However, their interval of nested cross-validation error estimates is almost identical. Our conclusion would be to use ridge regression for cross-validation on both datasets, but the expected difference between PLS and ridge cross-validatory chosen models are minute.

**Figure 12:**
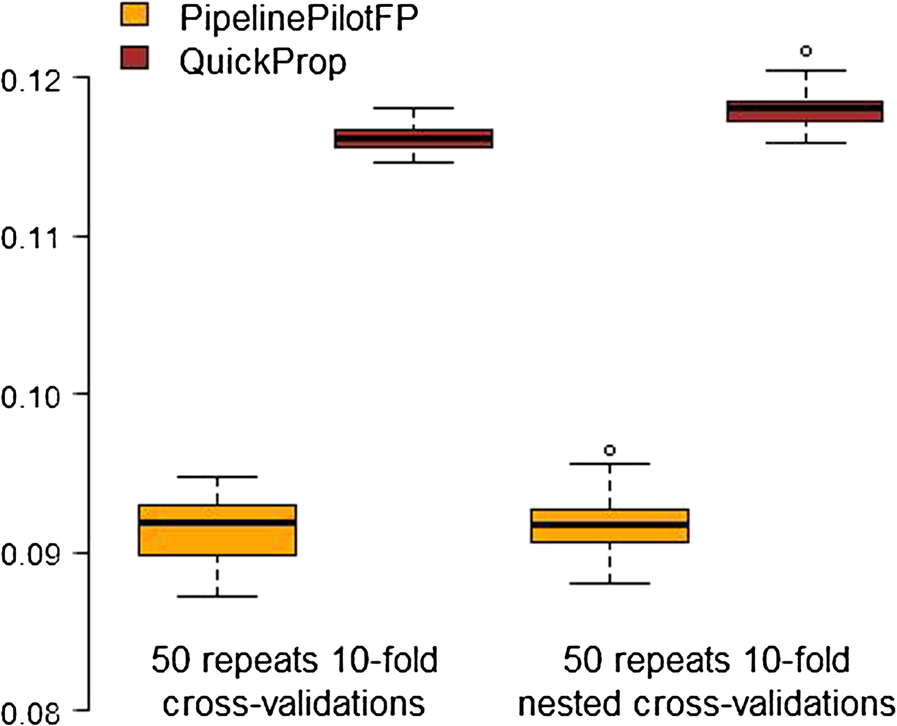
**Cross-validation and nested cross-validation proportion misclassified for ridge logistic regression on caco-PipelinePilotFP and caco-QuickProp.** Boxplots of 50 cross-validation and 50 nested cross-validation proportion misclassified for ridge logistic regression on caco-PipelinePilotFP and caco-QuickProp.

It is interesting that for caco-PipelinePilotFP nested cross-validation proportions misclassified are almost identical to those for cross-validation, while for caco-QuickProp they are slightly higher (Figure 12). Our conclusion is that if we would to use ridge logistic regression to predict caco and we had to chose between PipelinePilotFP and QuickProp descriptors, we would chose PipelinePilotFP.

**Figure 13:**
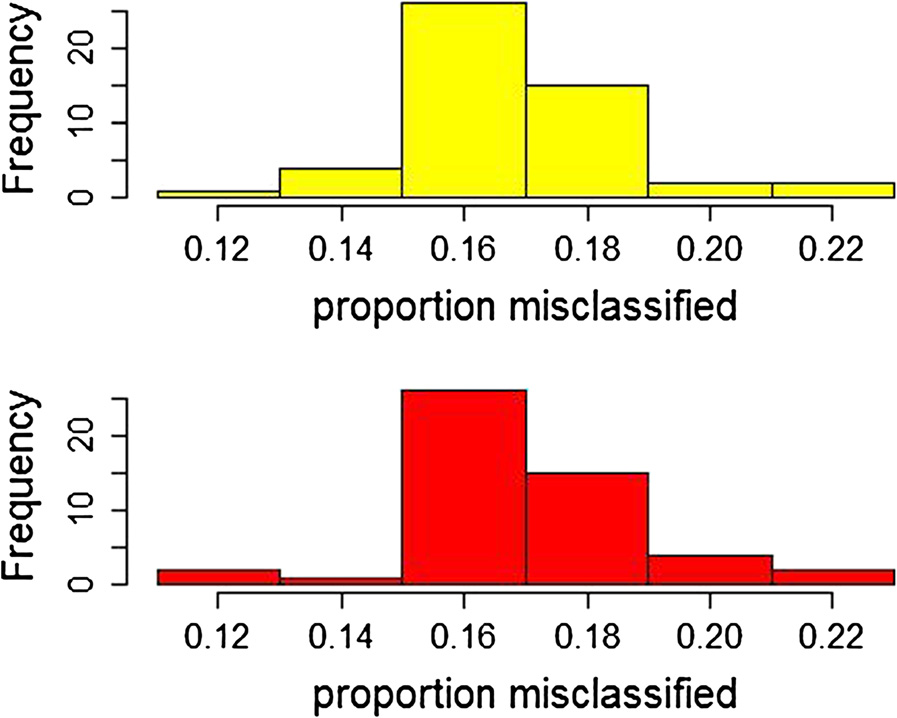
**Cross-validation vs nested cross-validation for ridge logistic regression on bbb2.** Histogram of 50 cross-validation and 50 nested cross-validation proportion misclassified for ridge logistic regression on bbb2.

**Figure 14:**
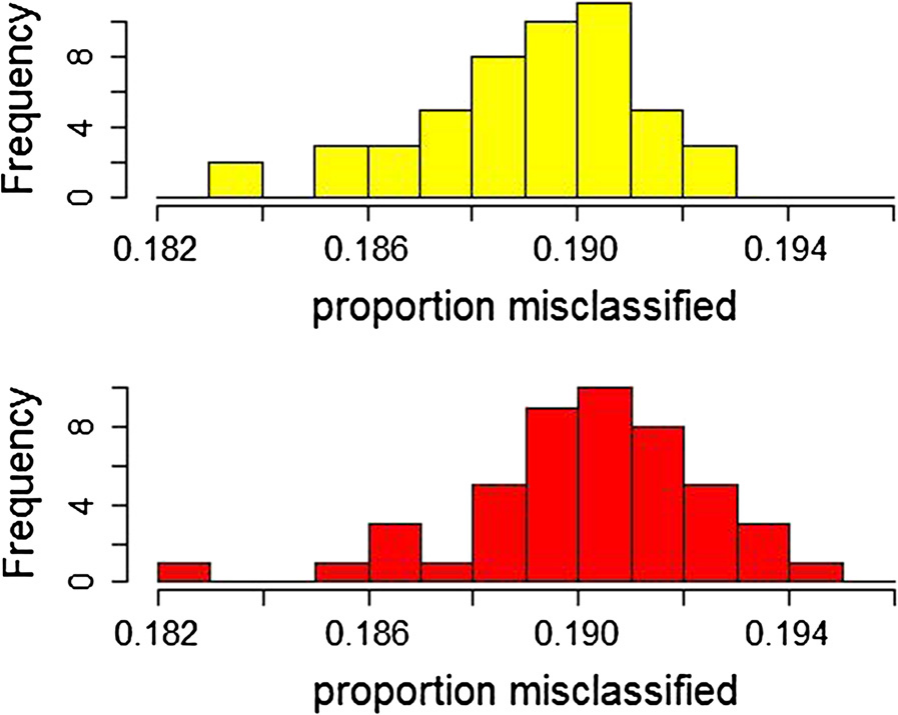
**Cross-validation vs nested cross-validation for ridge logistic regression on Mutagen.** Histogram of 50 cross-validation and 50 nested cross-validation proportion misclassified for ridge logistic regression on Mutagen.

**Figure 15:**
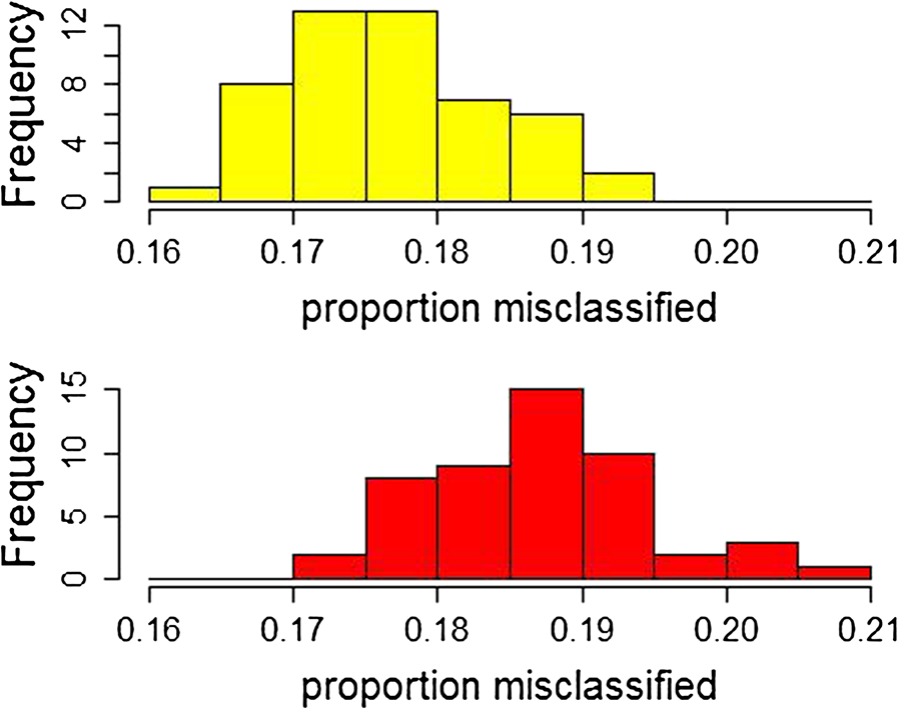
**Cross-validation vs nested cross-validation for ridge logistic regression on PLD.** Histogram of 50 cross-validation and 50 nested cross-validation proportion misclassified for ridge logistic regression on PLD.

In the cases of bbb2 (Figure 13), Mutagen (Figure 14) and PLD (Figure 15), where we only performed ridge logistic regression, the interval of nested cross-validation error estimates give us realistic expectations regarding our usage of the cross-validation protocol.

### Variable selection and parameter tuning

**Figure 16:**
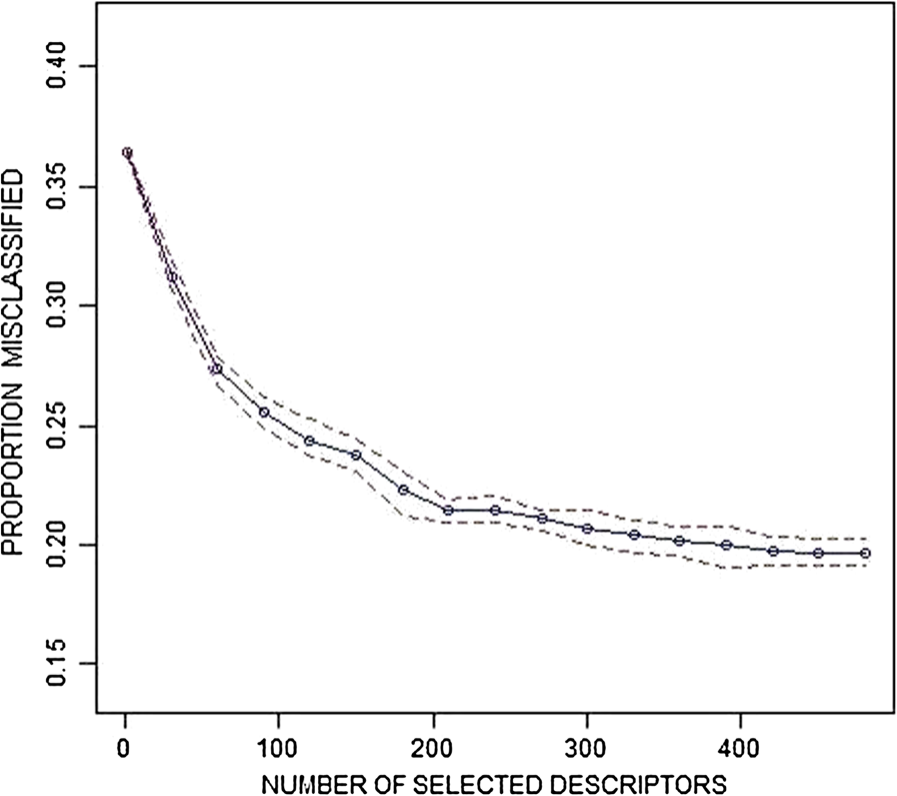
**Pearson’s rank based selection with linear SVM on Mutagen.** Minimum, mean and maximum cross-validated proportion misclassified from 50 repeats of 10-fold cross-validation of Pearson’s rank based selection with linear SVM on Mutagen.

As an example of Algorithm 3, we applied linear SVM coupled with Pearson’s rank based selection on the Mutagen dataset. We searched for the optimal number of descriptors from 1 to 480 with a step size of 30 {1, 30, 60, .. , 450, 480} using linearSVM with the following C parameters {0.5, 1, 2, 4, 8, 16}. Our grid search consisted of 21 × 6 = 126 points, and we repeated the cross-validation process 50 times. The minimum, mean and maximum cross-validated proportion misclassified from 50 repeats were calculated for all 126 grid points. In order to show results graphically, we selected the cost parameter which generated the lowest mean cross-validation error for each number of selected descriptors. Figure 16 shows the minimum, mean and maximum cross-validated proportion misclassified for every number of selected descriptors. The lowest average cross-validated misclassification error (0.196) is found for n = 450 and C = 8. In other words, this approach selected 450 descriptors, using Pearson’s rank based selection procedure, and linear SVM model with C = 8 as the classifier.

## Discussion

We sought to analyse and improve upon the existing cross-validation practices in selection and assessment of regression and classification models. No single cross-validation run provided for reliable selection of the best model on those datasets. Robust model selection required summarising the loss function across multiple repetitions of cross-validation. The model selection behaviour of a particular dataset could only be discerned upon performing the repeated cross-validation. Our model selection was based on average loss.

The nested cross-validation loss estimates differed significantly compared with the cross-validation estimates of the best model on at least caco-QuickProp, Melting Point Mutagen and PLD datasets. This confirms previous reports in the literature (Varma and Simon [9]).

Model assessment using repeated nested cross-validation (Figures 10, 11, 12, 13, 14 and 15) showed large variation of loss estimates across the nested cross-validation runs. For example, the proportion misclassified estimate for bbb2 varied between approximately 0.13 and 0.23 (Figure 13). In practical terms, this means that the best model selected on this dataset may have large-sample performance of anywhere between 13% and 23%. Whether this is adequate for a particular application is a domain-dependent question, however we point out that the repeated nested cross-validation provides the means to make an informed decision regarding the acceptance of the best model.

In all our examples we used 10-fold cross-validation. Kohavi [6] and Hastie *et al.*[4] empirically show that V-fold cross-validation compared to leave-one-out cross-validation has lower variance, and therefore tends to select simpler models. For some examples we executed 5-fold cross-validation and 5-fold nested cross-validation (results not shown), but did not observe a substantial difference from 10-fold.

Table 3 shows the summary of optimal cross-validatory chosen models for all nine datasets. When reporting the chosen parameter it is important to specify the details of the protocol, i.e. number of folds in cross-validations, the grid width and size, as well as the number of repeats.

We mentioned previously that Dudoit and van der Laan [8] proved the asymptotics of the cross-validatory choice for V-fold cross-validation. However, Breiman *et al.*[25] have found in the case of selecting optimal tree size for classification tree models that the tree size with minimal cross-validation error generates a model which generally overfits. Therefore, in Section 3.4.3 of their book Breiman *et al.*[25] define the one standard error rule (1 SE rule) for choosing an optimal tree size, and they implement it throughout the book. In order to calculate the standard error for single V-fold cross-validation, accuracy needs to be calculated for each fold, and the standard error is calculated from V accuracies from each fold. Hastie *et al.*[4] define the 1 SE rule as selecting the most parsimonious model whose error is no more than one standard error above the error of the best model, and they suggest in several places using the 1 SE rule for general cross-validation use. The main point of the 1 SE rule, with which we agree, is to choose the simplest model whose accuracy is comparable with the best model. However, when we repeat cross-validations standard error becomes smaller and the 1 SE rule does not have any effect. We are proposing that the rule needs to be redefined in the repeated cross-validation context.

There are situations where the practitioner just needs to find an optimal model and the issue of its assessment is not important. In those cases, it is not necessary to perform nested cross-validation. However, in most practical applications with limited sample sizes, use of predictive models depends on reliable model assessment. As far as we are aware, nested cross-validation is the best non-parametric approach for model assessment when cross-validation is used for model selection. As we have mentioned before, nested cross-validation estimate is not a property of the selected model, but rather comprises assessment of the selected model M *and* the protocol P used to select it. To reflect this fact, we introduced notation *P-estimate* to refer to nested cross-validation estimate of the large-sample performance of model M. As an example, consider cross-validation of linear SVM with three cost values and five folds (protocol P1) vs. cross-validation with seventeen cost values and five folds (protocol P2), and assume the minimum error rate is achieved by the same model M (i.e., same cost) in both experiments. The corresponding nested cross-validations will, in general, yield two different P-estimates (P1-estimate and P2-estimate, respectively) of the model M performance. This is reflection of the fact that the two cross-validations scanned different regions of hyper-parameter space, and the two P-estimates reflect different information incorporated into the selected best model. Thus, the P-estimates of the selected model differ, since they describe performance of different (model, protocol) pairs. We argue that this characteristic of nested cross-validation does not detract from its’ utility, however it is critically important to recognize it in order to properly interpret the results.

In the past, the major concern with grid search was that it was either computationally infeasible or very expensive. However, with the advent of cloud computing, new concern is that its extensive use in cross-validation will generate statistical models which will overfit in practice. Here we need to separate two issues:

1. How we define the optimisation problem for minimising cross-validation error estimates?
2. How we solve the optimisation problem?

As Dudoit and van der Laan [8] have shown, the first question is well defined with larger samples in V-fold cross-validation. However, Breiman et al. [25] have shown that cross-validatory chosen models are too complex in their examples. In the literature this issue is known as the *cross-validation bias*[26]. We are aware of three systematic approaches to solving this problem in the cross-validation context:

1. 1 SE rule as suggested by Breiman *et al.* [25] and Hastie *et al.* [4]
2. Corrected V-fold cross-validation as suggested by Burman [27]
3. Penalised V-fold cross-validation as suggested by Arlot [28].

Once we define an optimisation target, i.e. find parameters which minimise cross-validation error estimate, our aim is to find the optimal solution. Grid search is not the only systematic approach to hyper parameter optimisation. Recently Bergstra and Bengio [29] gave the case for using random search, while in the past we used Nelder and Mead [30] method. Regardless of the search method we use, the goal is to find the optimal parameter. We suggest using grid search because it is simple to implement and its parallelisation in the cloud is trivial. In our practice we prefere dense grids and the answer to question how dense is usually related to the costs.

In our findings, sparse grids do not necessarily lead to simpler models nor reduced overfitting. In all our examples where we applied PLS with grid being number of components from 1 till 60 with step 1. If we had chosen a less dense grid with number of components from 5 till 60 with step 5, then on AquaticTox the cross-validatory chosen number of components would be 15 (instead of 13 as with original dense grid), while on MeltingPoint the cross-validatory chosen number of components would be 50 (instead of 47 as with original dense grid). As the consequence of using such a less dense grid, our cross-validatory chosen model on both datasets would be more complex than the original dense grid.

It is important to note that both Stone [2] and Varma and Simon [9] use leave-one-out cross-validation, while we use V-fold cross-validation. The beauty of the leave-one-out cross-validation is that it generates the same results each time it is executed, and there is no need to repeat it. So it is possible to execute only single leave-one-out cross-validation and single nested leave-one-out cross-validation. However, as we have pointed out earlier, leave-one-out tends to select models with higher variances, which lead to overfitting, and for that reason we use V-fold cross-validation.

The computational cost is usually mentioned as the main drawback of nested cross-validation. In our examples, we repeat 50 times 10-fold nested cross-validation which means that for nine examples we performed 500 times full model selection process, where each model selection consists of 50 times repeated 10-fold cross-validation. Various authors proposed simplifications which obviate the need for the extensive computations. Tibshirani and Tibshirani [31] propose a bias correction for the minimum error rate in cross-validation which does not require additional computation. Bernau *et al.*[32] suggest another correction which would reduce the computational costs associated with nested cross-validation. We propose that the computational cost of performing repeated cross-validation and nested cross-validation in the cloud have reached a level where the use of substitutes to full nested cross-validation are no longer justified.

In discovery research projects there are experimental costs associated with the training samples. At a certain point in the project, the following question is usually asked: Will the additional data improve our predictive models and, if so, by how much? If the samples are generated randomly from the same population, then additional data will always improve the predictive model. However, the question is whether the additional costs of performing experiments will pay off in improvements to the model. In our opinion, here we can see the practical value of nested cross-validation. In case of Mutagen dataset, or even caco-PipelinePilotFP, where intervals of nested cross-validation errors are narrow and similar to cross-validations’, we can conclude that if we randomly remove 10% of samples, the quality of models remains almost the same. So we can say that additional increase of 10% of sample size will not significantly improve our current models.

Our results show that repetition is an essential component of reliable model assessment based on nested cross-validation. Any single nested cross-validation run cannot be used for assessing the error of an optimal model, because of its variance. We demonstrated the use of repeated nested cross-validation in order to get an interval of the estimate.

Furthermore, we demonstrated that there are datasets (for example, AquaticTox) where the interval of nested cross-validation errors is wide, and in which cases the user must assess the suitability of the model for the task in hand. We think that these situations point to the inadequacy of the dataset itself, rather than inadequacy of the nested cross-validation method. In such cases the application of repeated nested cross-validation points to the need to collect additional samples/compounds and/or alternative descriptors.

## Conclusions

Selection and assessment of predictive models require repeated cross-validation and nested cross-validation. The advent of affordable cloud computing resources makes these methods widely accessible. In our opinion, the ability to economically use large amounts of computer power over the cloud changes the perception of what is feasible and what is necessary to perform when selecting and assessing models.

## Authors’ original submitted files for images

Below are the links to the authors’ original submitted files for images. Authors’ original file for figure 1 Authors’ original file for figure 2 Authors’ original file for figure 3 Authors’ original file for figure 4 Authors’ original file for figure 5 Authors’ original file for figure 6 Authors’ original file for figure 7 Authors’ original file for figure 8 Authors’ original file for figure 9 Authors’ original file for figure 10 Authors’ original file for figure 11 Authors’ original file for figure 12 Authors’ original file for figure 13 Authors’ original file for figure 14 Authors’ original file for figure 15 Authors’ original file for figure 16 Authors’ original file for figure 17 Authors’ original file for figure 18 Authors’ original file for figure 19 Authors’ original file for figure 20 Authors’ original file for figure 21
