# Supplementary figures and images for: Cross-validation pitfalls when selecting and assessing regression and classification models
Source: J Cheminform. 2014 Mar 29;6:10. doi: 10.1186/1758-2946-6-10 (PMC3994246; doi:10.1186/1758-2946-6-10)

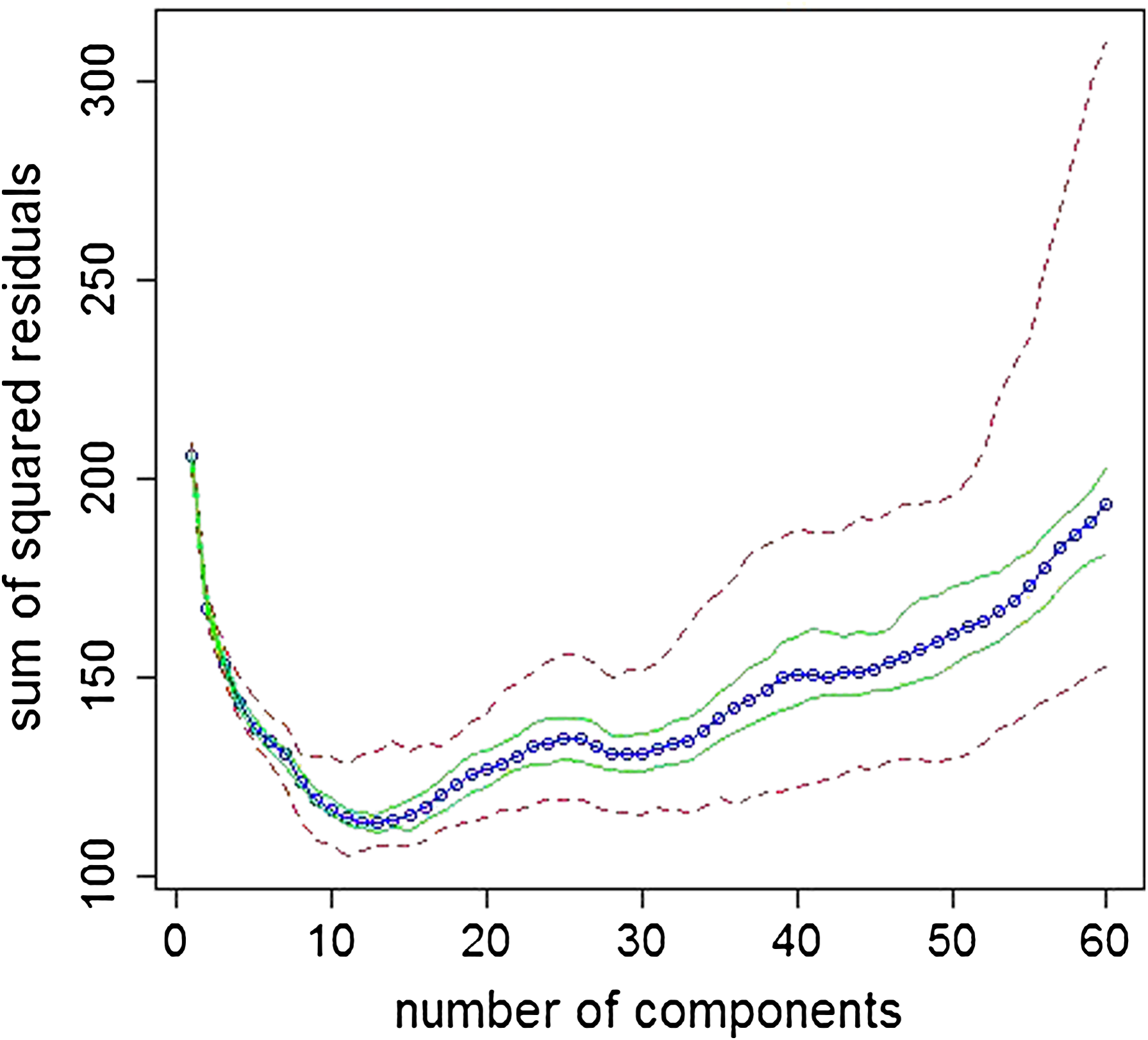

Supplement: Supplementary file 1 — Authors’ original file for figure 1 [file 13321_2014_587_MOESM1_ESM.tif]

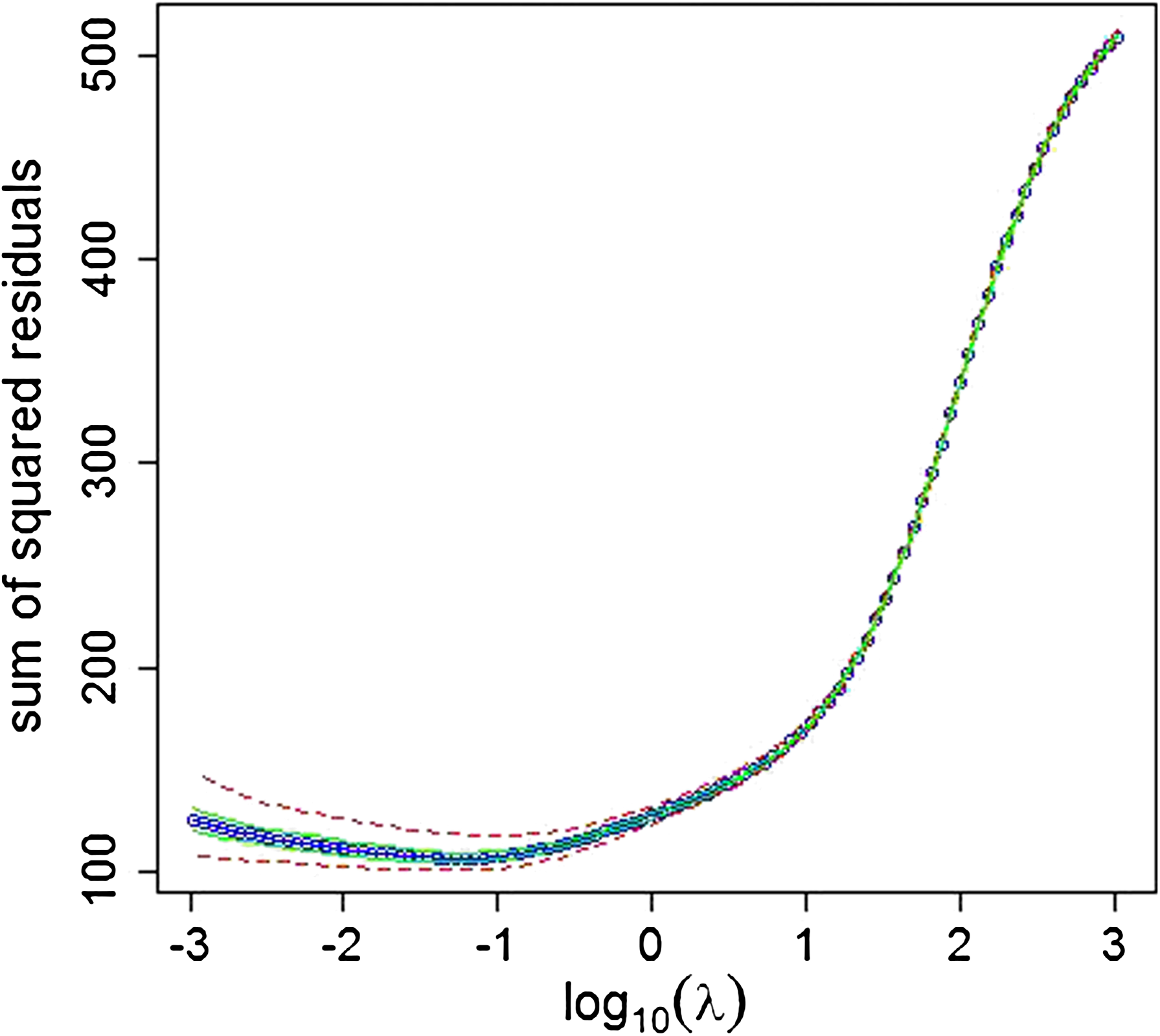

Supplement: Supplementary file 2 — Authors’ original file for figure 2 [file 13321_2014_587_MOESM2_ESM.tif]

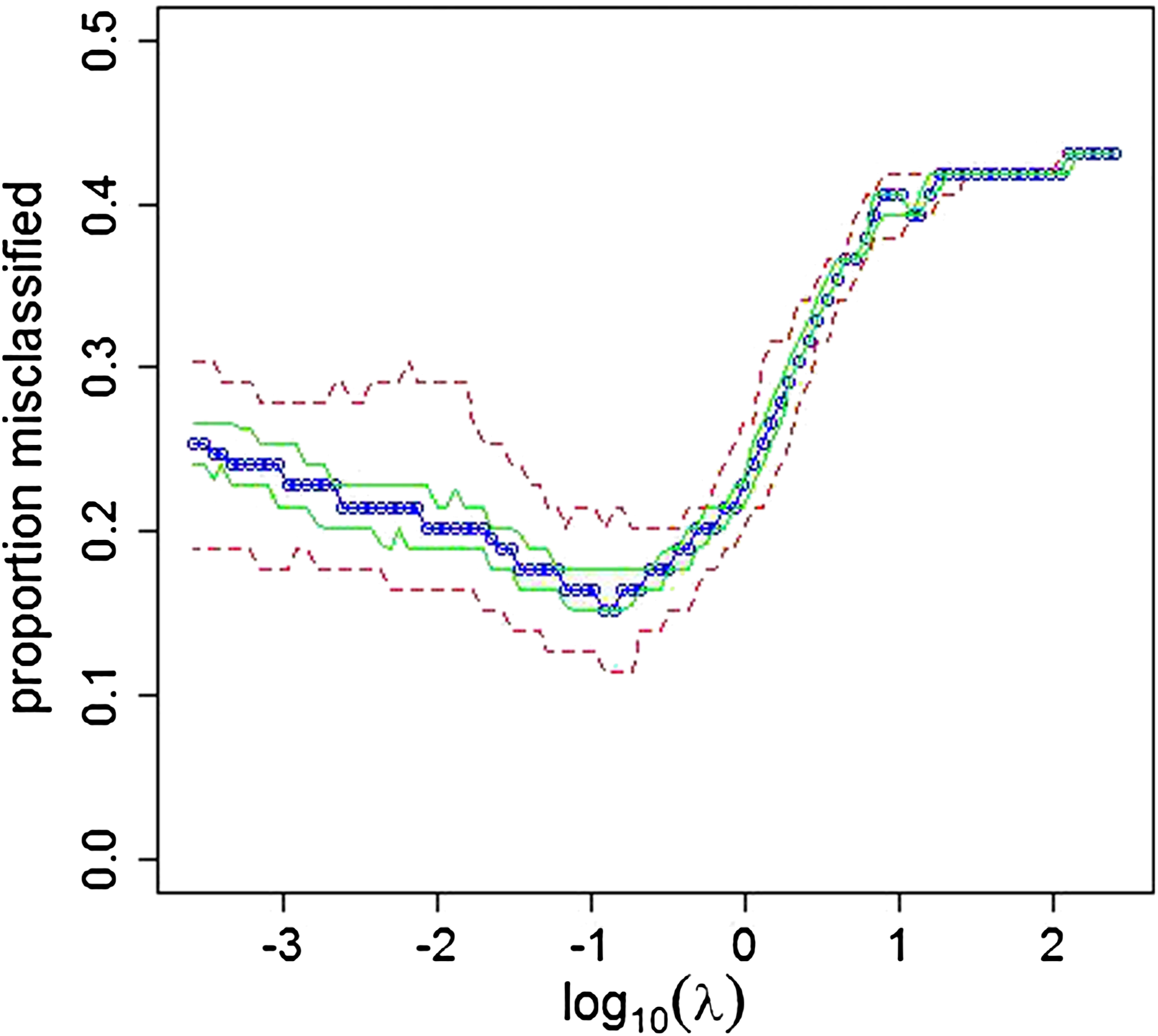

Supplement: Supplementary file 3 — Authors’ original file for figure 3 [file 13321_2014_587_MOESM3_ESM.tif]

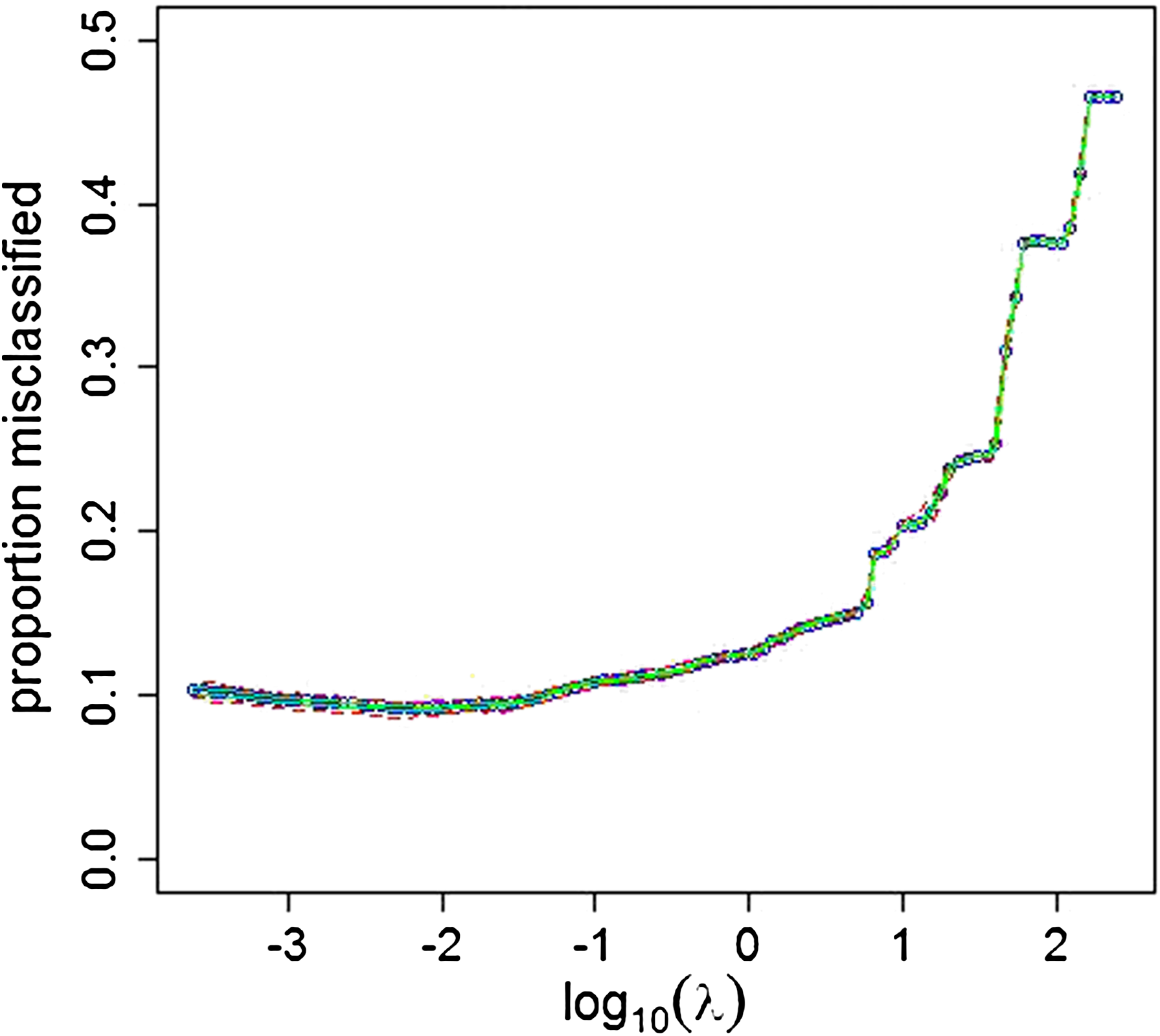

Supplement: Supplementary file 4 — Authors’ original file for figure 4 [file 13321_2014_587_MOESM4_ESM.tif]

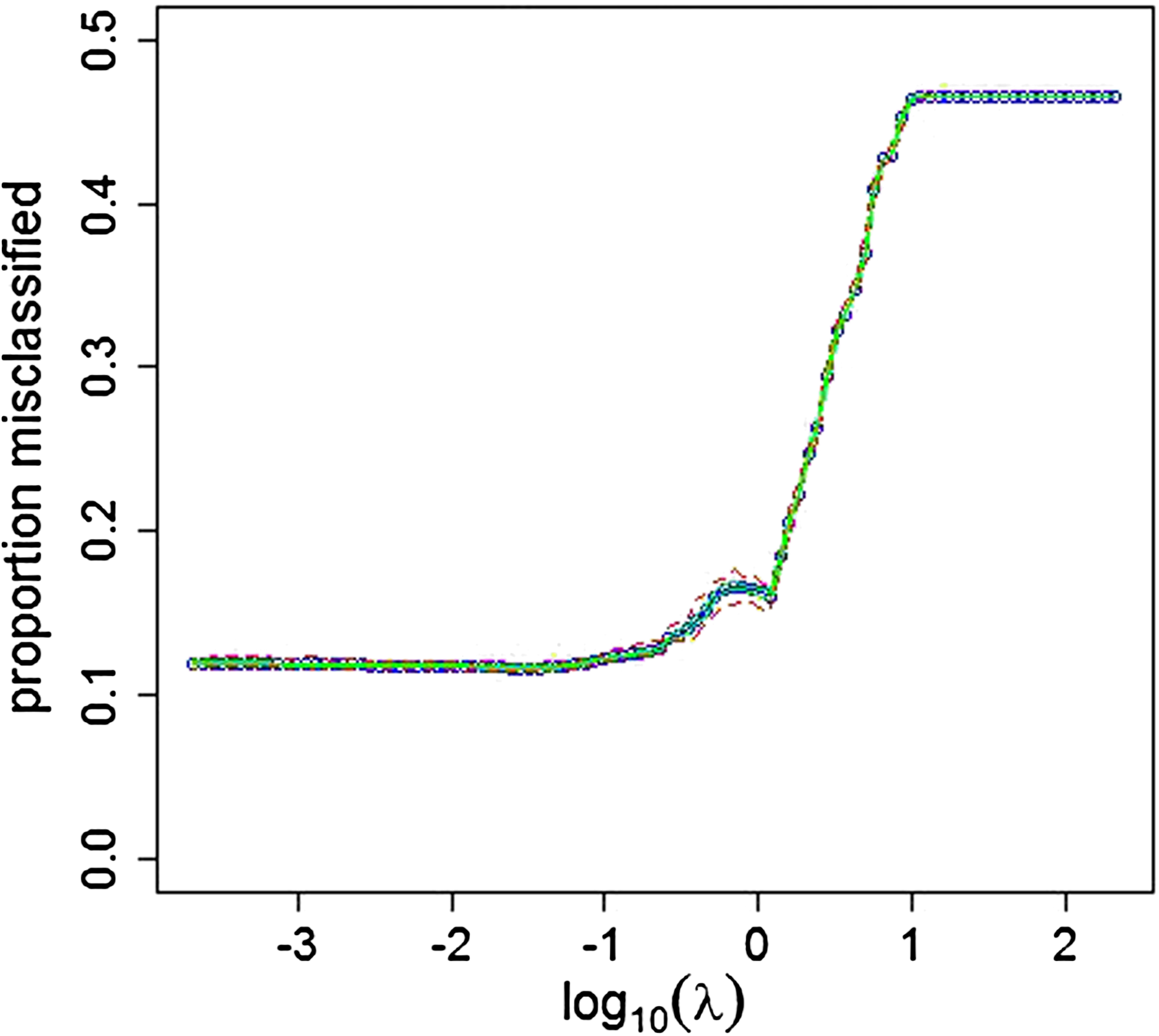

Supplement: Supplementary file 5 — Authors’ original file for figure 5 [file 13321_2014_587_MOESM5_ESM.tif]

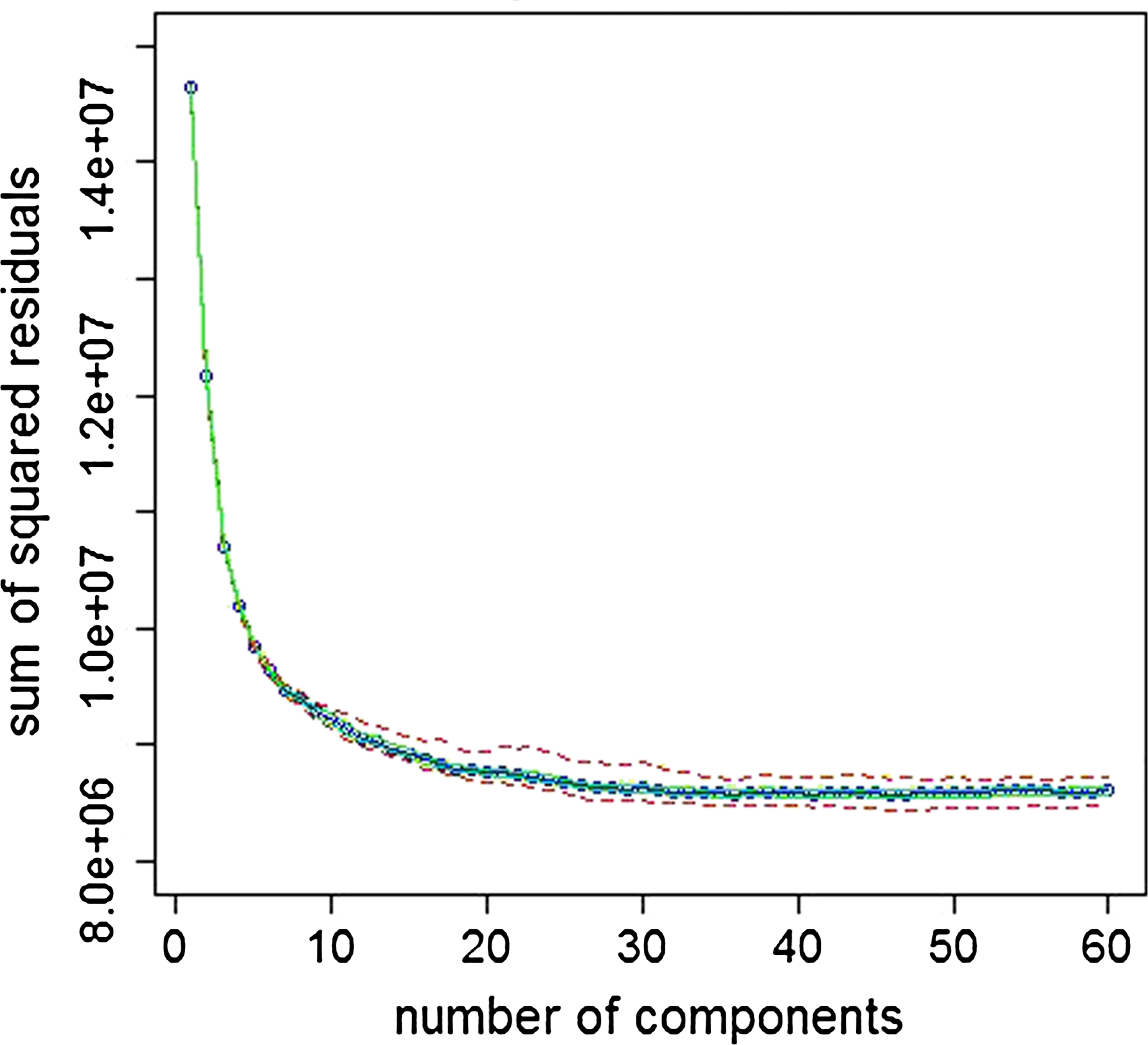

Supplement: Supplementary file 6 — Authors’ original file for figure 6 [file 13321_2014_587_MOESM6_ESM.tif]

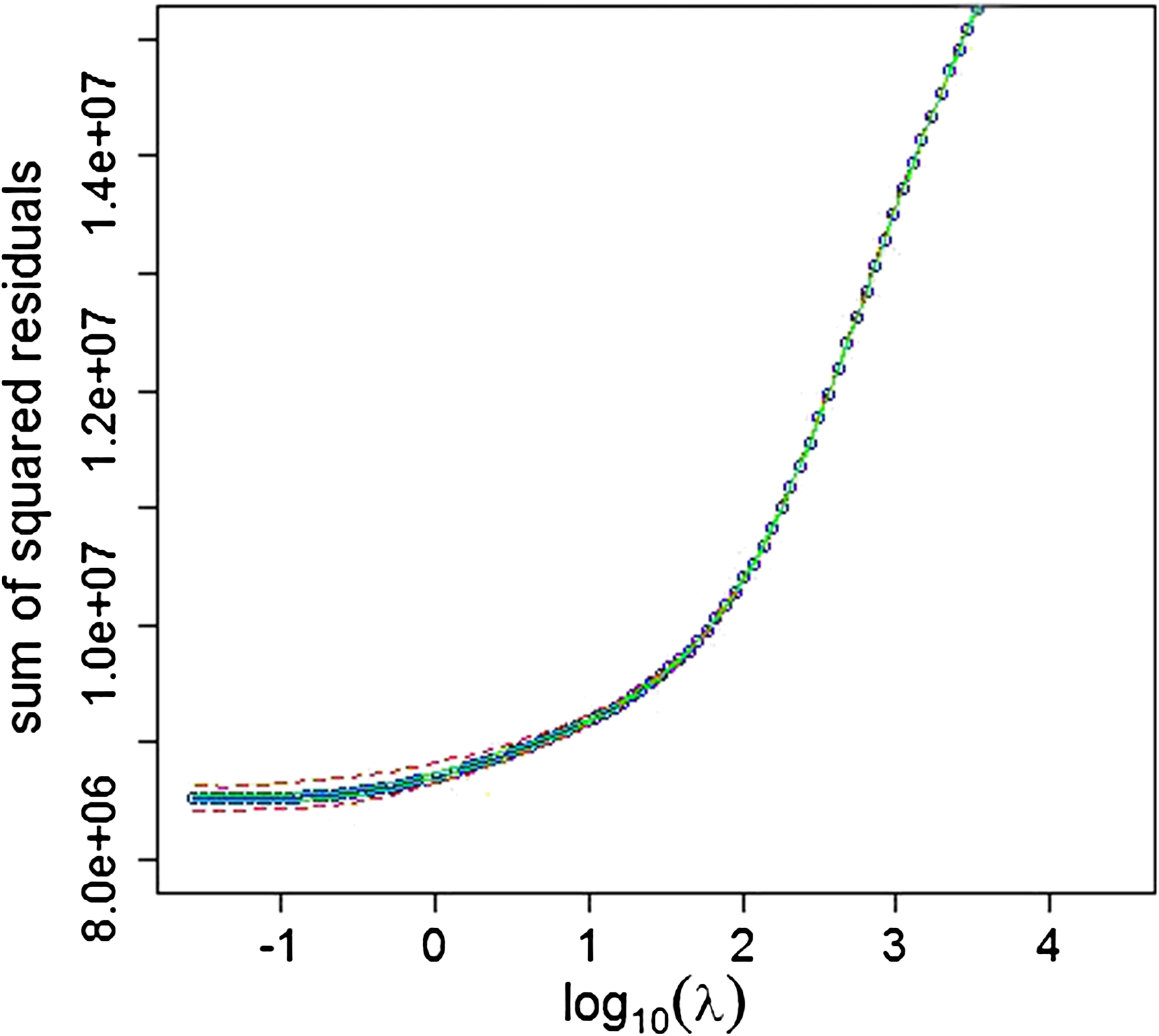

Supplement: Supplementary file 7 — Authors’ original file for figure 7 [file 13321_2014_587_MOESM7_ESM.tif]

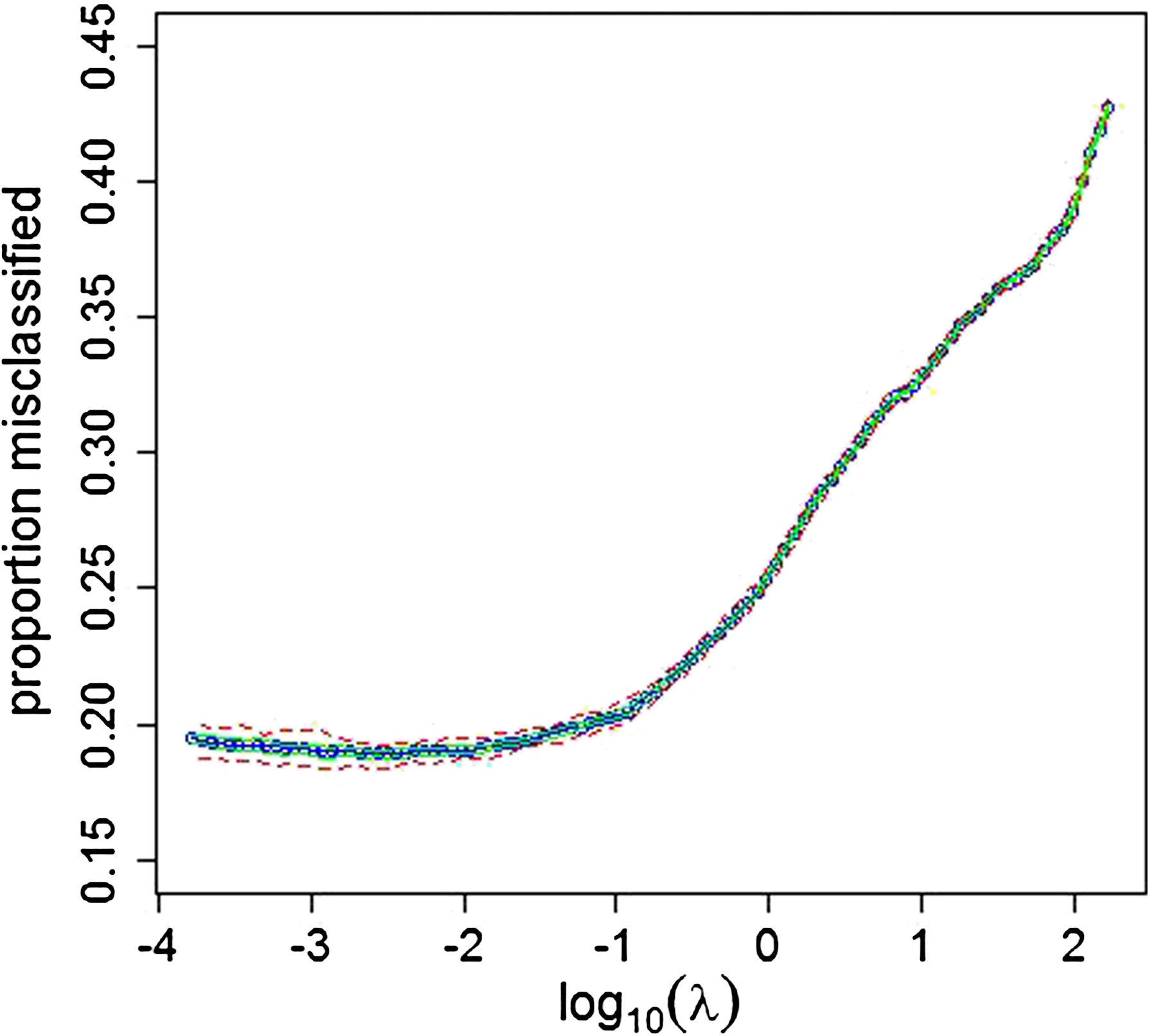

Supplement: Supplementary file 8 — Authors’ original file for figure 8 [file 13321_2014_587_MOESM8_ESM.tif]

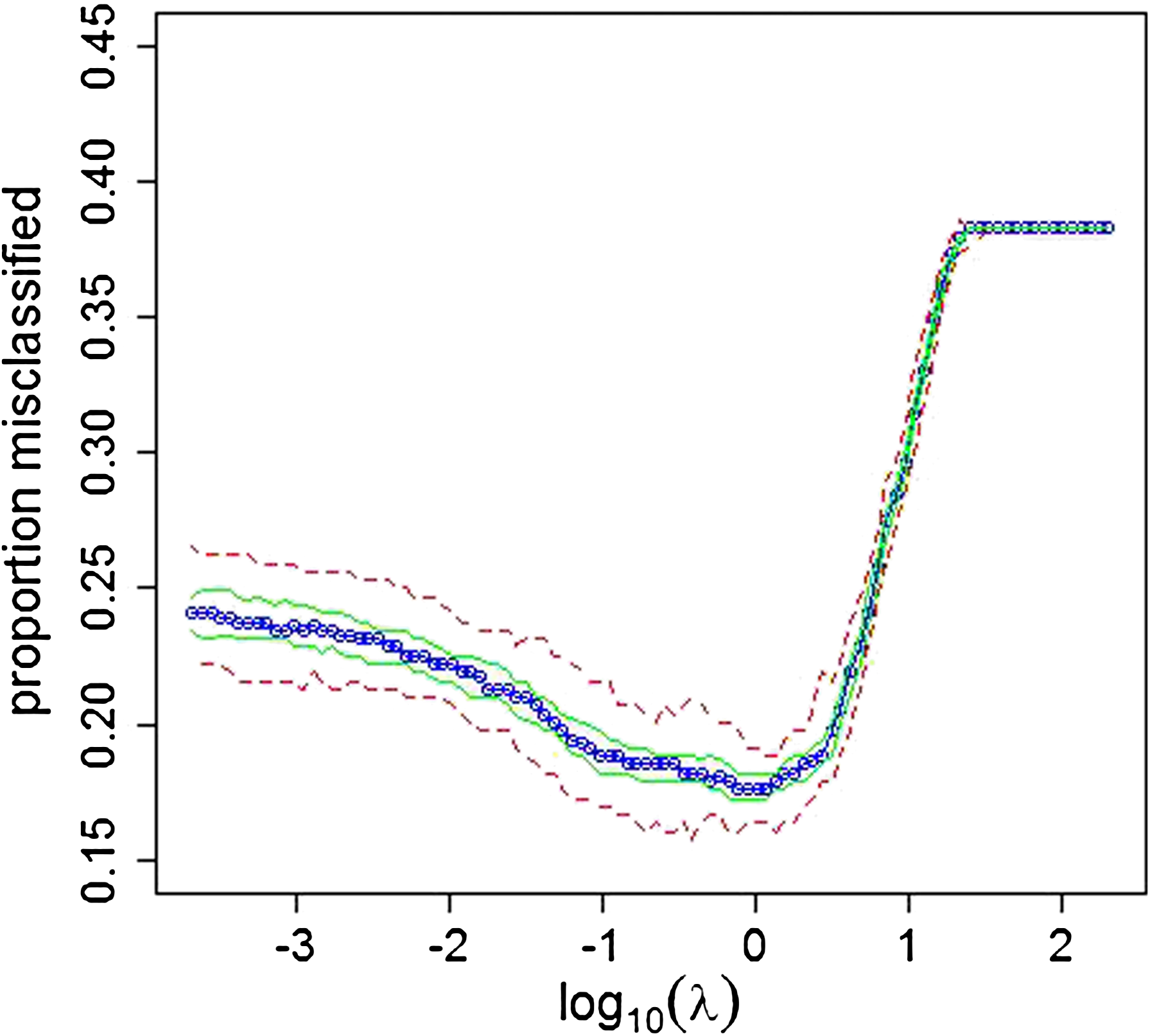

Supplement: Supplementary file 9 — Authors’ original file for figure 9 [file 13321_2014_587_MOESM9_ESM.tif]

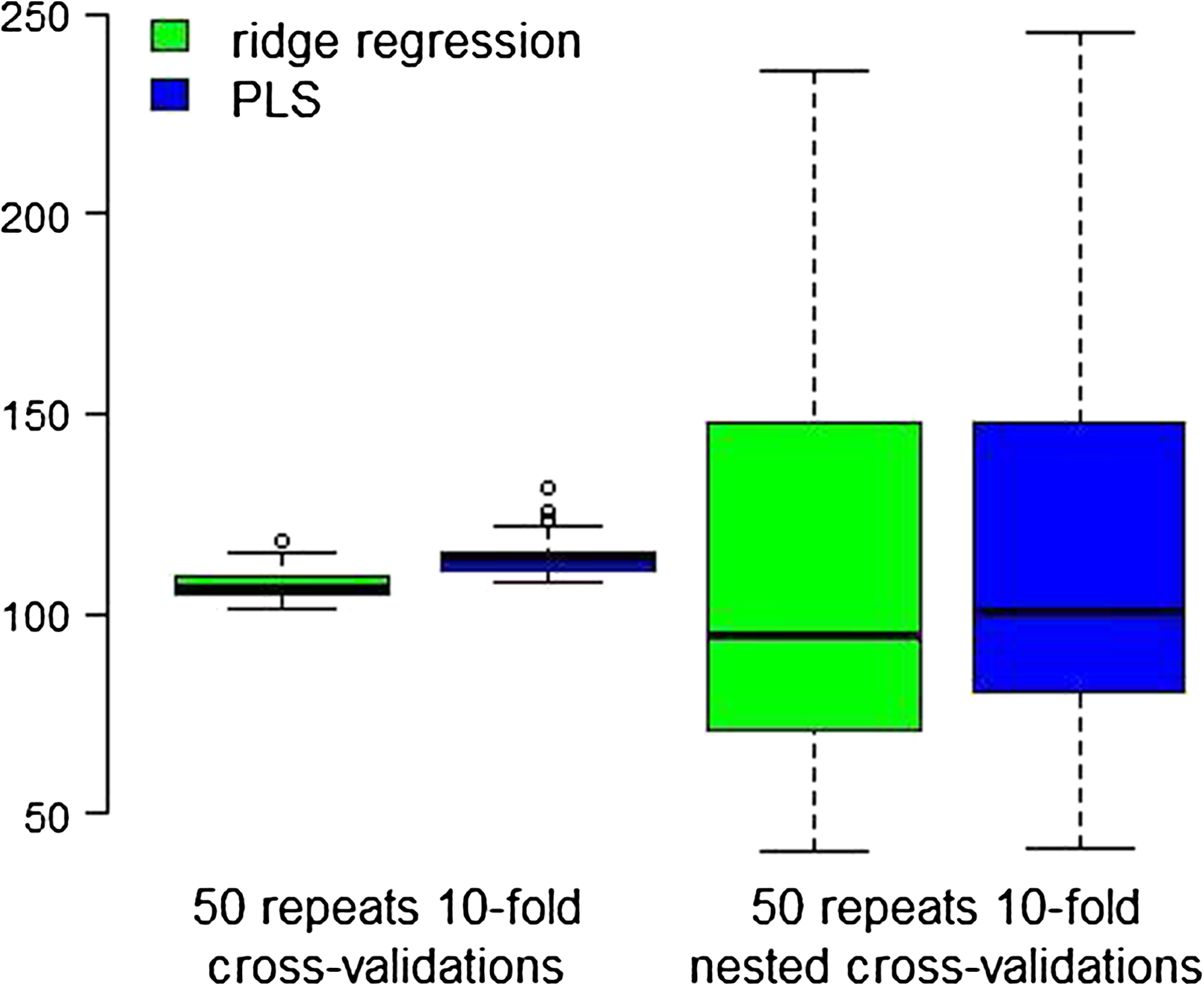

Supplement: Supplementary file 10 — Authors’ original file for figure 10 [file 13321_2014_587_MOESM10_ESM.tif]

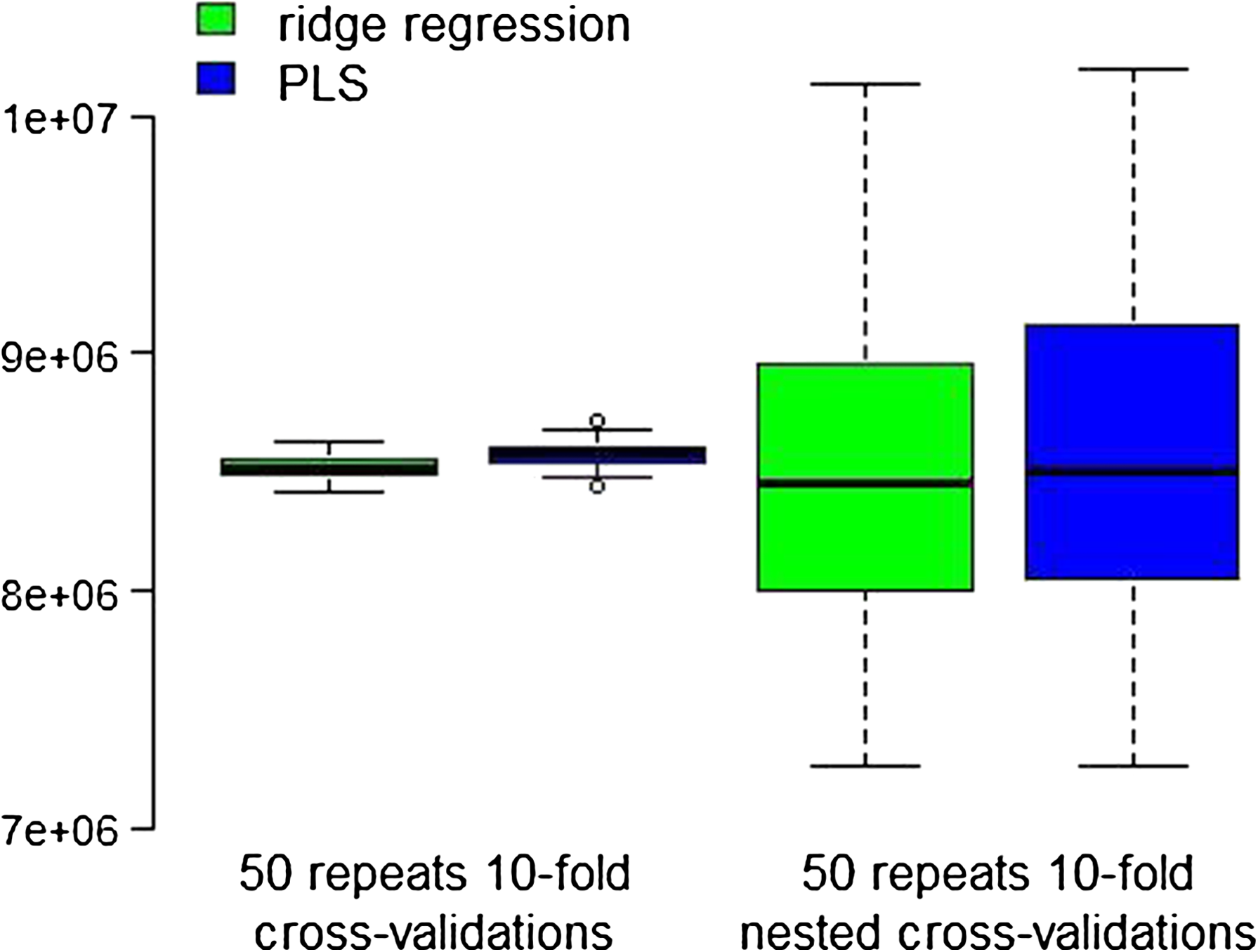

Supplement: Supplementary file 11 — Authors’ original file for figure 11 [file 13321_2014_587_MOESM11_ESM.tif]

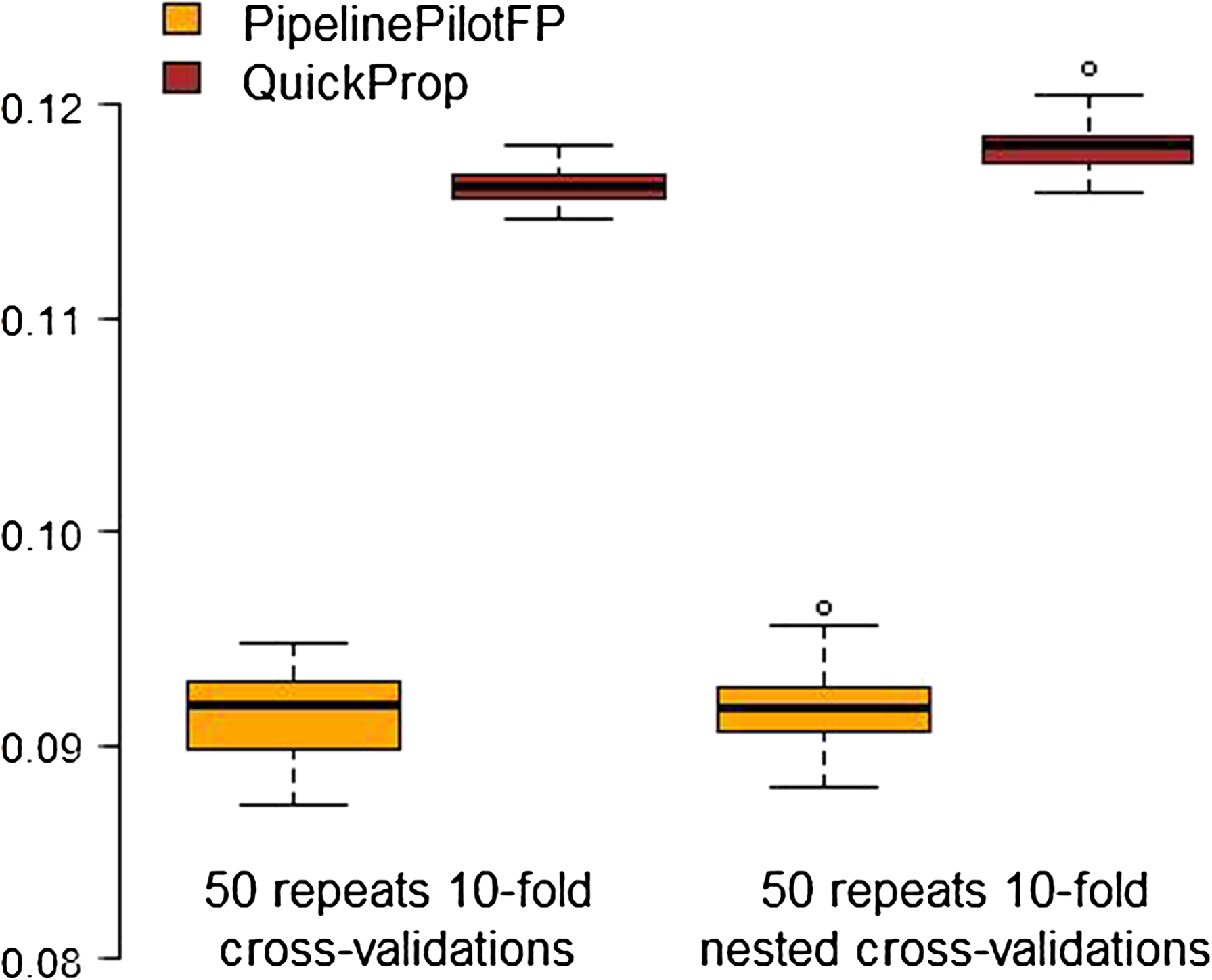

Supplement: Supplementary file 12 — Authors’ original file for figure 12 [file 13321_2014_587_MOESM12_ESM.tif]

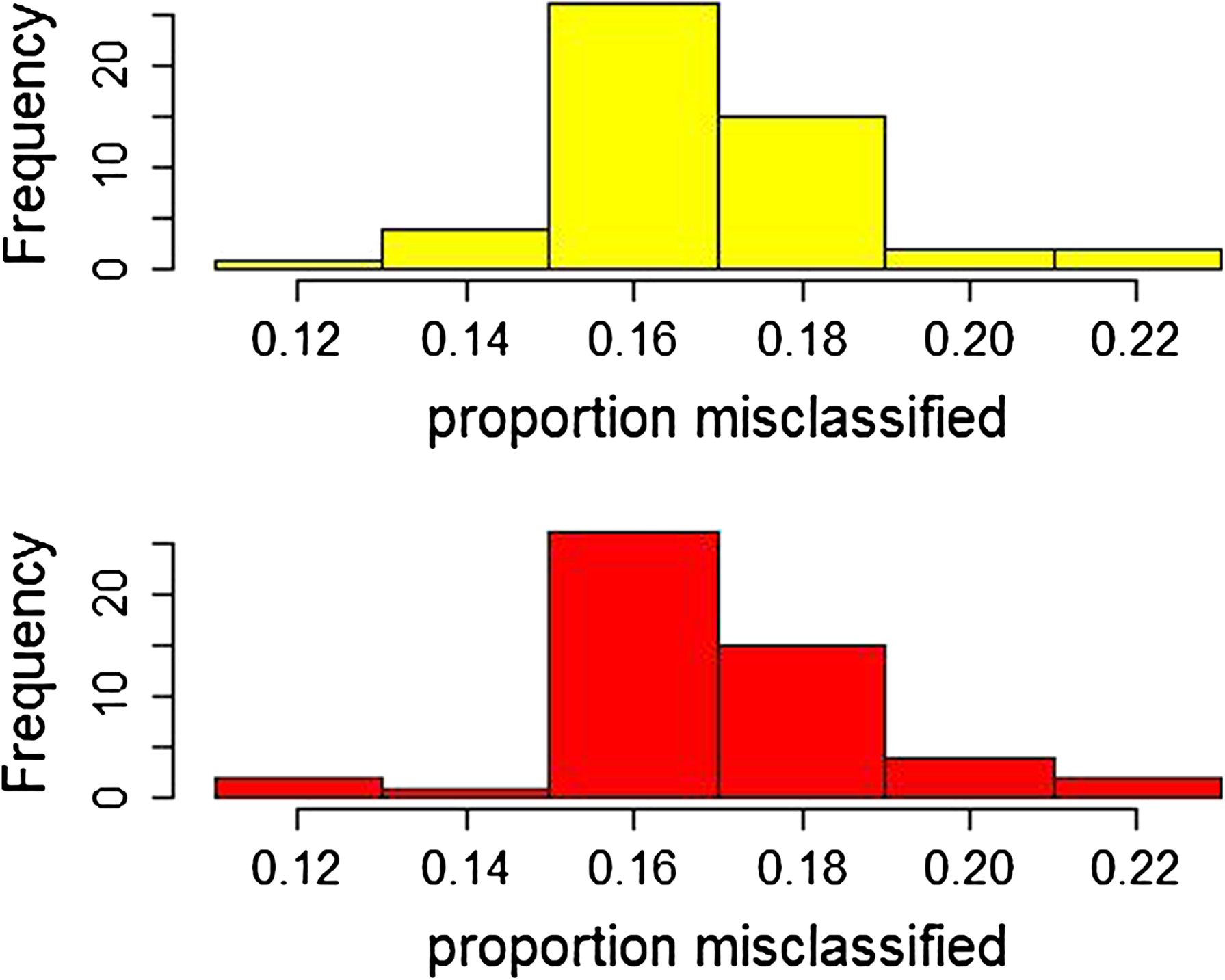

Supplement: Supplementary file 13 — Authors’ original file for figure 13 [file 13321_2014_587_MOESM13_ESM.tif]

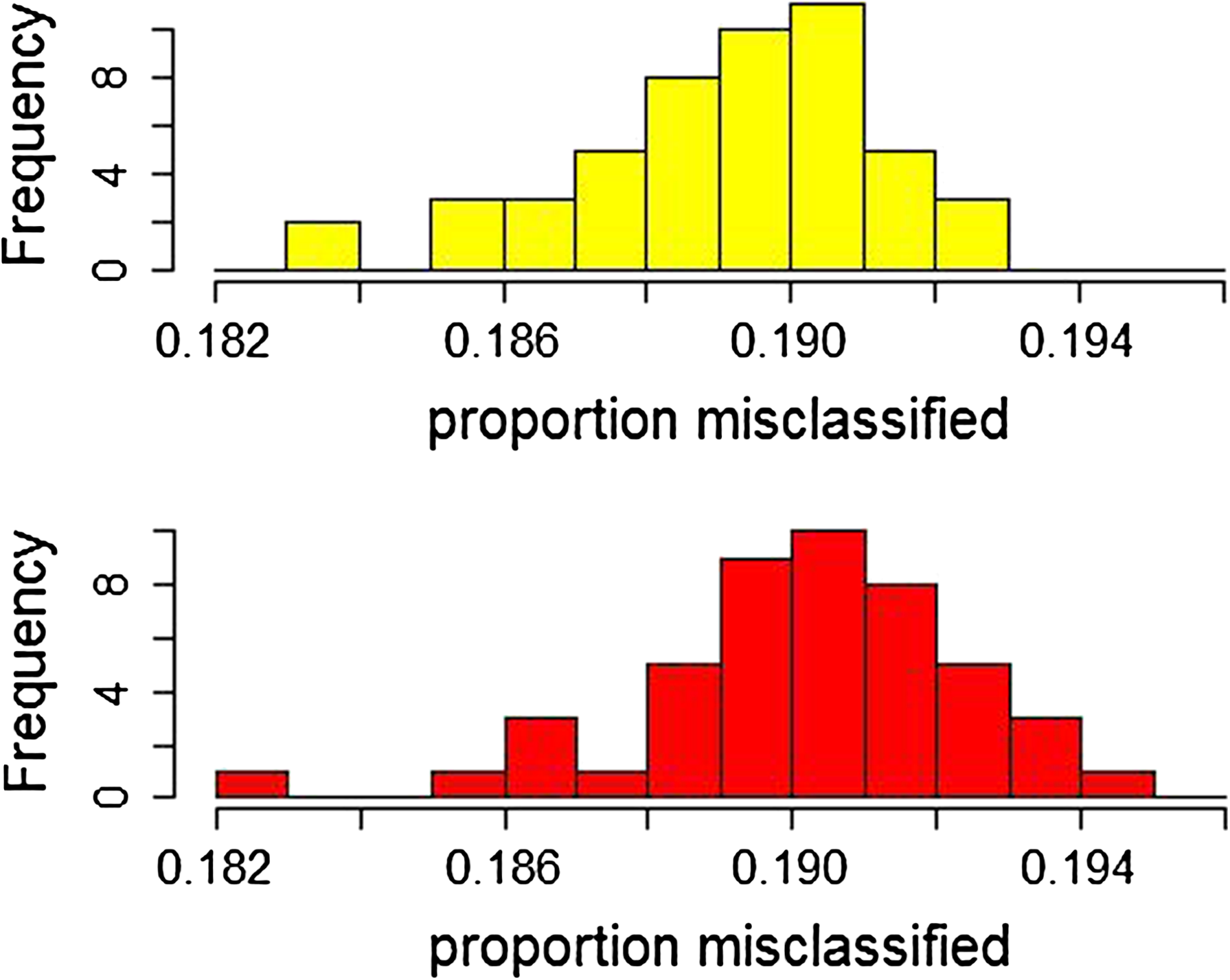

Supplement: Supplementary file 14 — Authors’ original file for figure 14 [file 13321_2014_587_MOESM14_ESM.tif]

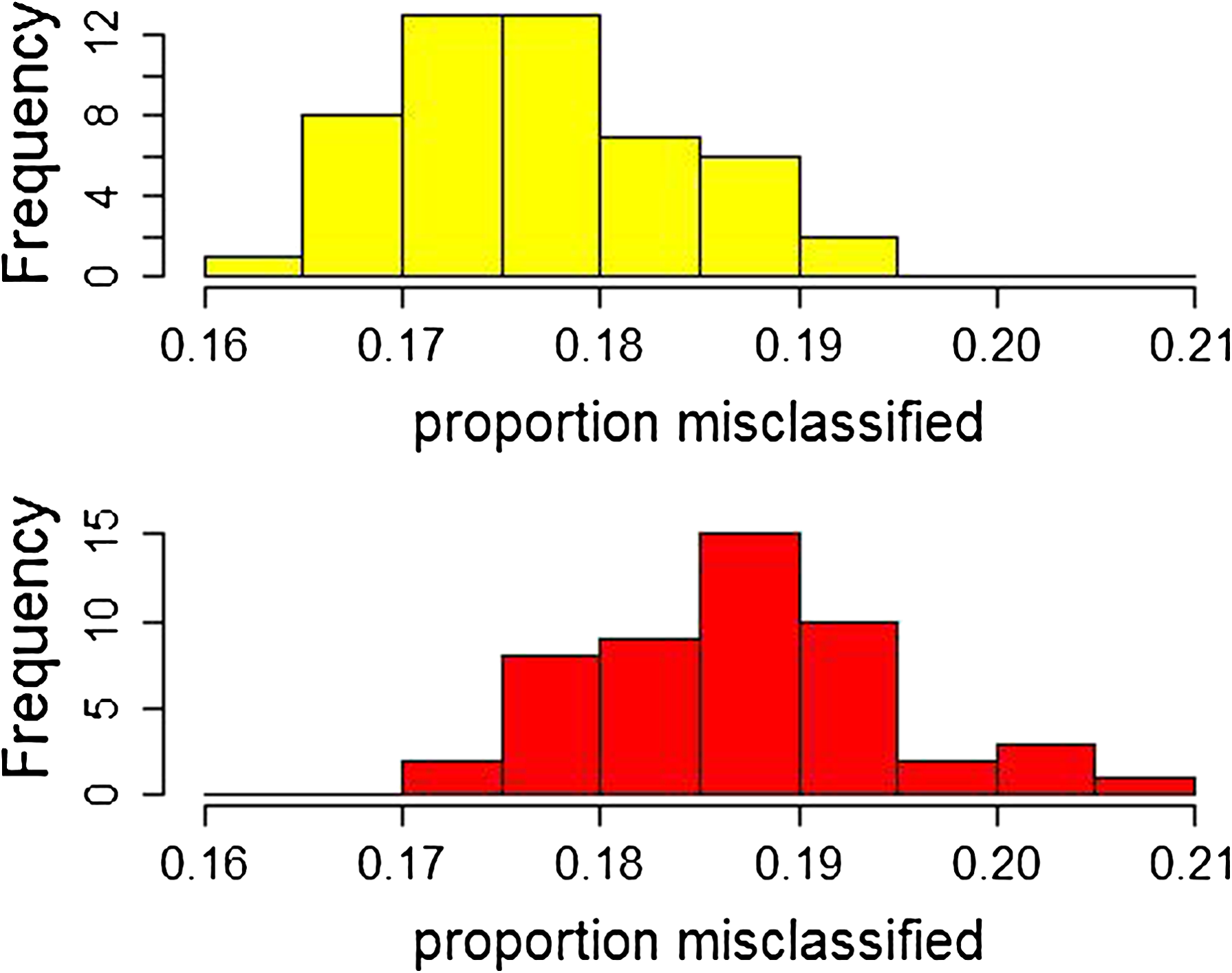

Supplement: Supplementary file 15 — Authors’ original file for figure 15 [file 13321_2014_587_MOESM15_ESM.tif]

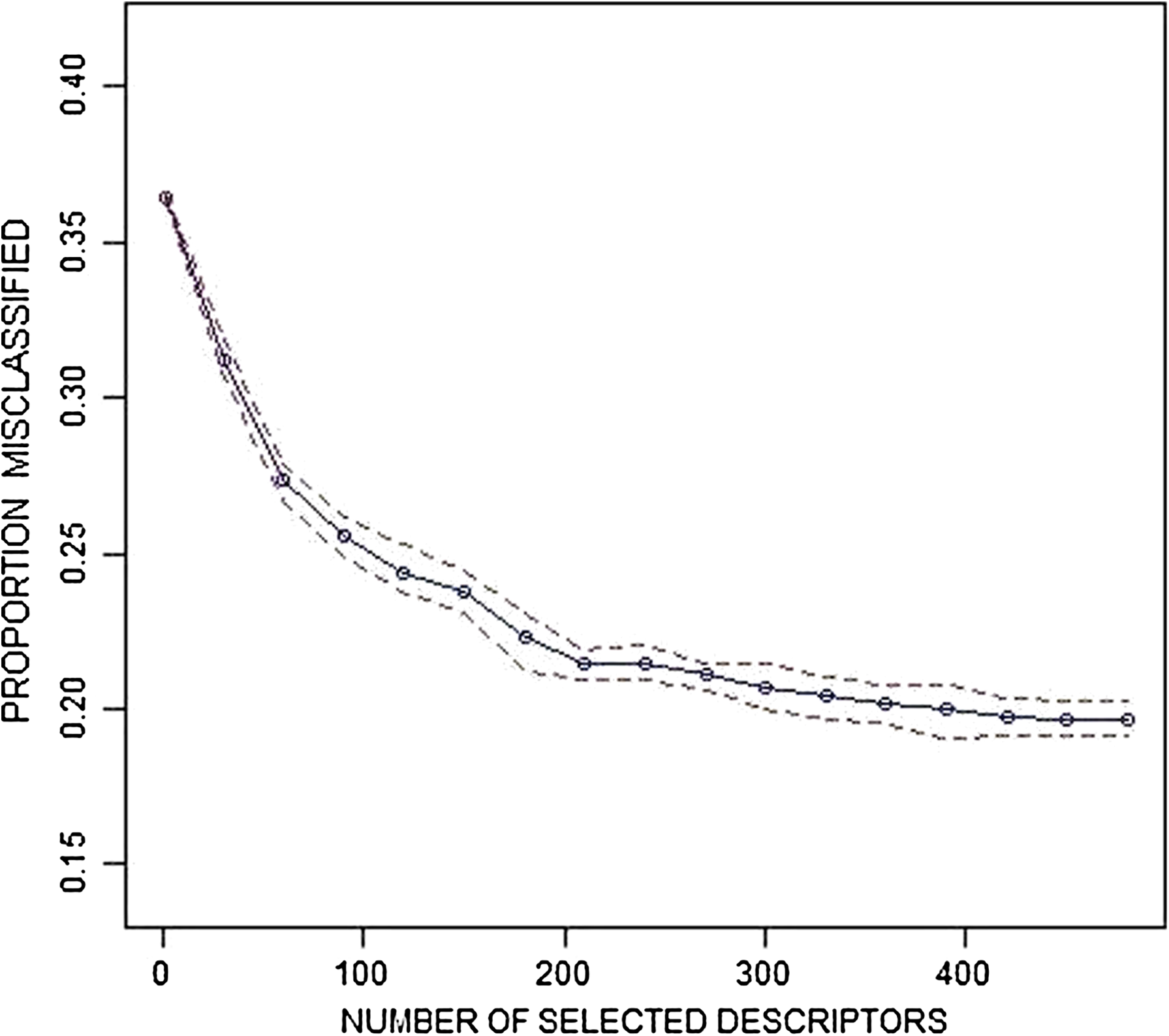

Supplement: Supplementary file 16 — Authors’ original file for figure 16 [file 13321_2014_587_MOESM16_ESM.tif]

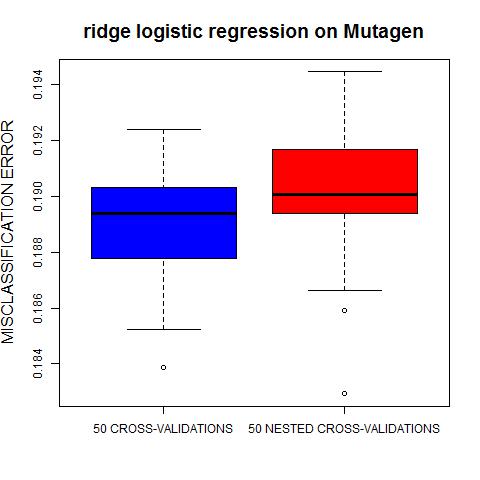

Supplement: Supplementary file 17 — Authors’ original file for figure 17 [file 13321_2014_587_MOESM17_ESM.jpeg]

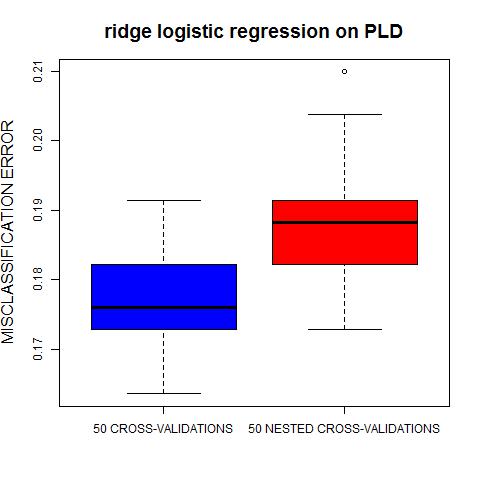

Supplement: Supplementary file 18 — Authors’ original file for figure 18 [file 13321_2014_587_MOESM18_ESM.jpeg]

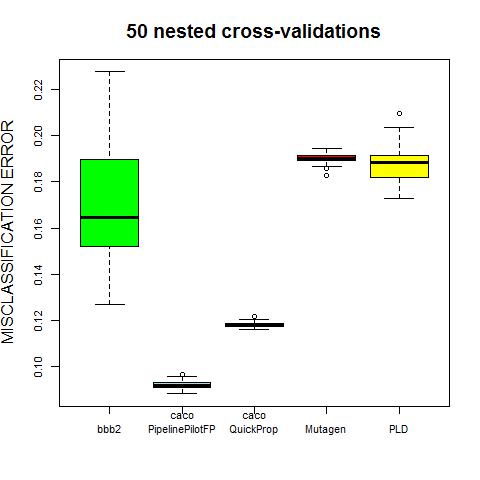

Supplement: Supplementary file 19 — Authors’ original file for figure 19 [file 13321_2014_587_MOESM19_ESM.jpeg]

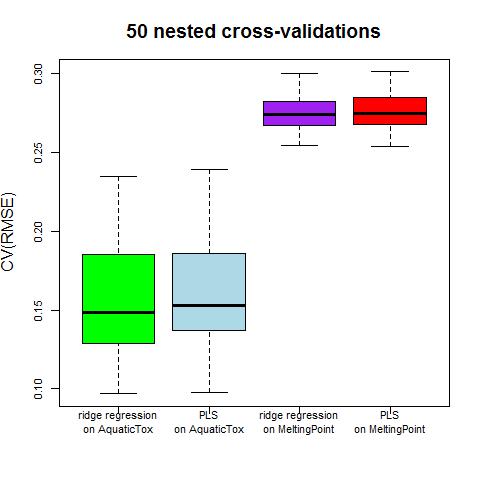

Supplement: Supplementary file 20 — Authors’ original file for figure 20 [file 13321_2014_587_MOESM20_ESM.jpeg]

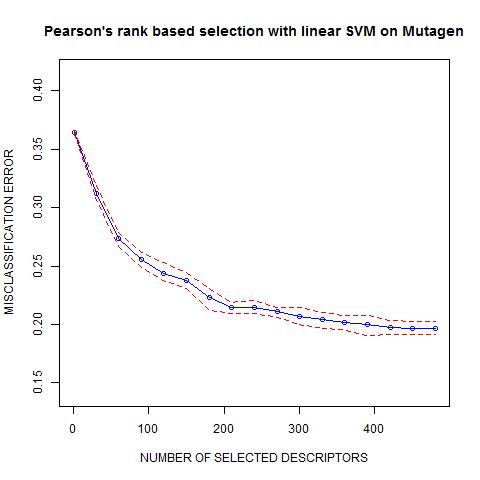

Supplement: Supplementary file 21 — Authors’ original file for figure 21 [file 13321_2014_587_MOESM21_ESM.jpeg]
